# Supplementary material for: Modulation of transcription burst amplitude underpins dosage compensation in the Drosophila embryo
Source: Cell Rep. 2023 Apr 14;42(4):112382. doi: 10.1016/j.celrep.2023.112382 (PMC10283159; doi:10.1016/j.celrep.2023.112382)
Supplement: Document S2. Article plus supplemental information [file mmc11.pdf]

Cell Reports

Article

Modulation of transcription burst amplitude underpins dosage compensation in the *Drosophila* embryo

Graphical abstract

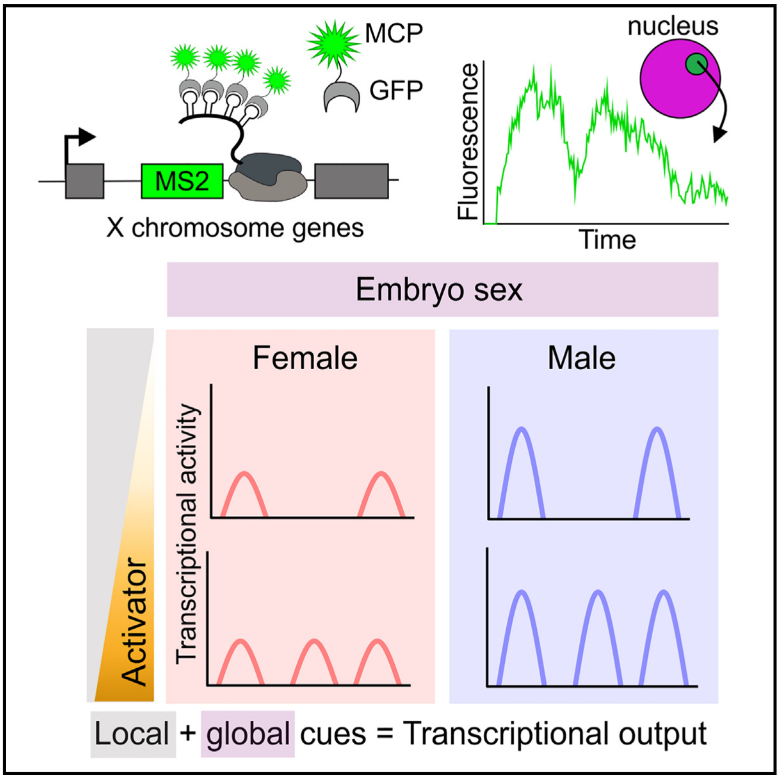

Authors

Lauren Forbes Beadle, Hongpeng Zhou, Magnus Rattray, Hilary L. Ashe

Correspondence

magnus.rattray@manchester.ac.uk (M.R.),
hilary.ashe@manchester.ac.uk (H.L.A.)

In brief

Using live imaging to study transcription of dosage-compensated X chromosome genes at single-cell resolution, Forbes Beadle et al. show that transcriptional bursts have higher amplitude in male embryos compared with females. This gender cue is integrated with transcription factor concentration, which regulates burst frequency, to establish the transcriptional response.

Highlights

- MS2 imaging of transcription of dosage-compensated genes at single-cell resolution
- X chromosome genes undergo transcriptional bursting in male and female embryos
- Dosage-compensated X chromosome genes have higher burst amplitude in male embryos
- X chromosome gene transcription is regulated spatially by changes in burst frequency

Forbes Beadle et al., 2023, Cell Reports *42*, 112382
April 25, 2023 © 2023 The Authors.
https://doi.org/10.1016/j.celrep.2023.112382

CellPress

Cell Reports

CellPress
OPEN ACCESS

Article

Modulation of transcription burst amplitude underpins dosage compensation in the *Drosophila* embryo

Lauren Forbes Beadle,[1] Hongpeng Zhou,[1] Magnus Rattray,[1,*] and Hilary L. Ashe[1,2,*]
[1]Faculty of Biology, Medicine and Health, University of Manchester, Manchester M13 9PT, UK
[2]Lead contact
*Correspondence: magnus.rattray@manchester.ac.uk (M.R.), hilary.ashe@manchester.ac.uk (H.L.A.)
https://doi.org/10.1016/j.celrep.2023.112382

SUMMARY

Dosage compensation, the balancing of X-linked gene expression between sexes and to the autosomes, is critical to an organism's fitness and survival. In *Drosophila*, dosage compensation involves hypertranscription of the male X chromosome. Here, we use quantitative live imaging and modeling at single-cell resolution to study X chromosome dosage compensation in *Drosophila*. We show that the four X chromosome genes studied undergo transcriptional bursting in male and female embryos. Mechanistically, our data reveal that transcriptional upregulation of male X chromosome genes is primarily mediated by a higher RNA polymerase II initiation rate and burst amplitude across the expression domain. In contrast, burst frequency is spatially modulated in nuclei within the expression domain in response to different transcription factor concentrations to tune the transcriptional response. Together, these data show how the local and global regulation of distinct burst parameters can establish the complex transcriptional outputs underpinning developmental patterning.

INTRODUCTION

Dosage compensation was originally discovered in *Drosophila*,[1] where it was found that males increase the transcription of most active X chromosome genes up to 2-fold.[2–4] In *Drosophila*, the most widely supported models of dosage compensation include a direct role for the male-specific lethal (MSL) complex, which targets the male X chromosome. This complex is composed of five proteins—MSL1–3, Maleless, and the males absent on the first (MOF) histone acetyltransferase—and two non-coding RNAs transcribed from the X chromosome, *RNA on the X* (*roX*) *1* and *roX2*. Dosage compensation is restricted to males as, based on an X:autosome ratio of 1, the sex-lethal RNA-binding protein accumulates in female embryos and represses the translation of *msl2* mRNAs.[5,6]

A favored model for targeting of the MSL complex is that it binds to high-affinity sites (HASs) on the X chromosome, which include *roX1* and *roX2*,[7,8] then spreads along the X chromosome to the bodies of active genes.[7–11] Recruitment of the MSL complex to these HASs requires the CLAMP transcription factor.[12] In the early embryo, CLAMP initially binds genome wide and recruits the MSL complex, before both become enriched at HASs on the male X chromosome.[13] However, recently, an alternative model has been proposed whereby MSL2 and the *roX* RNAs trap the MSL complex on the X chromosome to nucleate a compartment that is necessary for dosage compensation. Evidence for this model includes the finding that the *roX* RNAs and MSL2, via its intrinsically disordered C-terminal domain, form stable condensates.[14]

An alternative model for dosage compensation, the inverse dosage model, also exists. This model is based on the idea of genomic balance from studies of aneuploidy and polyploidy, where there is a negative correlation between gene expression and chromosomal dosage.[15] It posits that the single X chromosome in males results in altered stoichiometry and activity of multi-subunit complexes, such as those involved in gene regulation, which would result in an upregulation of the entire genome.[16,17] In this model, the MSL complex is not directly required for X chromosome transcriptional upregulation. Instead MSL targeting to the X sequesters MOF and other histone modifiers away from the autosomes to mute their transcriptional upregulation. An additional activity is also suggested to constrain male X chromosome transcription that could arise from the high levels of histone acetylation due to MOF.[15]

An RNA polymerase II (RNA Pol II) elongation-based mechanism has been proposed to explain the doubling of transcription on the male X chromosome. This "jump start and gain" model is based on nascent RNA sequencing and RNA Pol II chromatin immunoprecipitation-on-chip (ChIP-chip) data from tissue culture cells, which revealed an MSL complex-dependent enhancement of RNA Pol II on gene bodies, particularly the 3′ ends. Elevated elongation was postulated to be a result of enhanced release of RNA Pol II from 5′ pausing, the "jump," and improved RNA Pol II processivity, the "gain."[18,19] As MOF within the MSL complex acetylates H4K16 predominantly on the X chromosome,[20–23] this modification was proposed to reduce the steric hindrance of

This is an open access article under the CC BY license (http://creativecommons.org/licenses/by/4.0/).

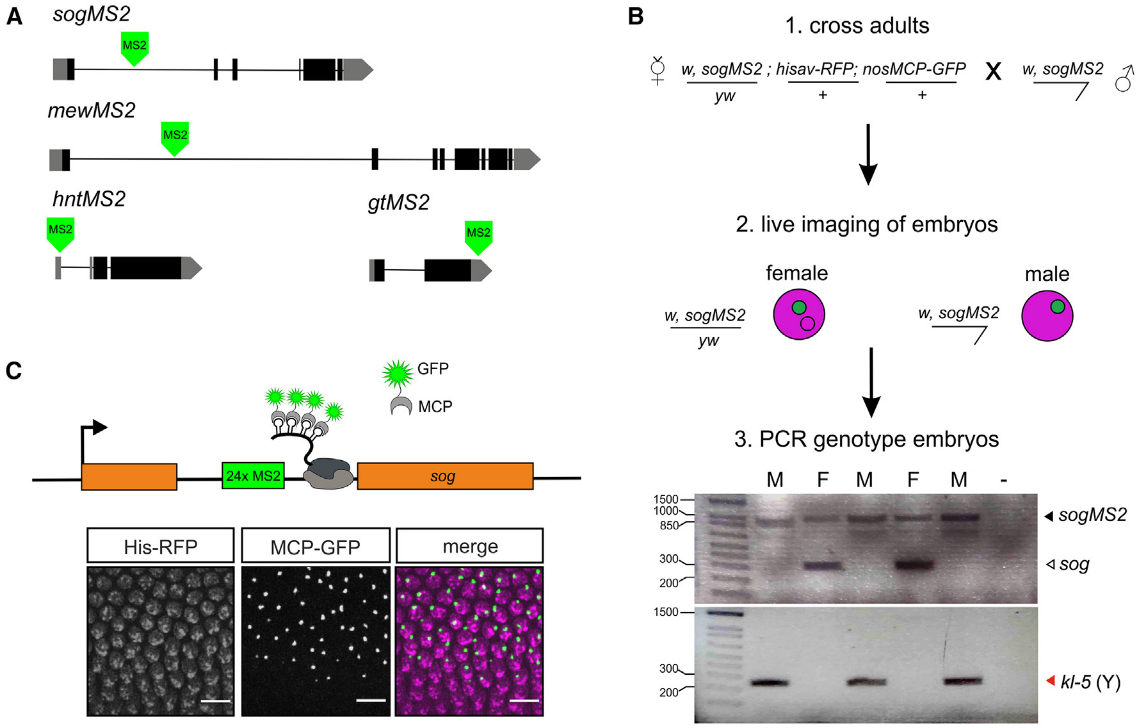

Figure 1. Live imaging of dosage-compensated transcription in the early embryo

(A) Schematics showing the position of the 24xMS2 loops in each of the four genes used for live imaging. The *hntMS2* and *gtMS2* lines have been described previously.[30,31]

(B) Overview of the imaging and embryo sexing pipeline. The cartoon shows a female embryo with a single fluorescent transcription site (TS) as used for the analysis, although some female embryos imaged had 2 fluorescent TSs. The gels show representative results for PCR genotyping of individual biological replicate *sogMS2* embryos to identify their sex using *sog* primers flanking the 24xMS2 loops for the X chromosome and *kl-5* primers for the Y chromosome. Female embryos have two bands for the *sog* primers as they are heterozygous for the MS2 insertion (black arrowhead) and so have one unmodified *sog* locus (white arrowhead). PCR product sizes are 283 (*sog*) and 248 bp (*kl-5*), and full DNA ladder sizes can be found in the STAR Methods.

(C) Top: cartoon showing that active transcription is detected by MCP-GFP binding to the MS2 loops in the mRNA as RNA Pol II transcribes the gene. Bottom: a still from a live imaging movie corresponding to a region from the full field of view of a *sogMS2* embryo labeled with His-RFP (magenta) and the nascent TSs marked by MCP-GFP fluorescence (green). The border of the expression domain is visible, showing active nuclei in the presumptive neuroectoderm and inactive nuclei in the mesoderm. Scale bar is 10 μm.

See also Figure S1.

nucleosomes to RNA Pol II.[18,19] However, an alternative initiation mechanism due to increased RNA Pol II recruitment has also been proposed based on a comparison of RNA Pol II ChIP sequencing (ChIP-seq) data from male, female, and MSL2 knockdown male salivary glands. Higher RNA Pol II was found at the promoters of a subset of genes on the wild-type male X,[24] although the relevance of the small (~1.2 fold) change in promoter RNA Pol II levels has been questioned.[25–27]

Advances in imaging have revealed that many genes are transcribed in discontinuous bursts of transcriptional activity in organisms ranging from bacteria to mammals.[28] In this study, we exploit live and quantitative imaging to determine whether hypertranscription of male X chromosome genes involves a higher frequency and/or amplitude of transcriptional bursts. Our data suggest that dosage compensation is mediated by a higher amplitude of transcriptional bursts in male embryos. In contrast, burst frequency is tuned locally in a sex-independent manner to coordinate the transcriptional output to local transcription factor inputs.

RESULTS

Live imaging of dosage-compensated transcription in the *Drosophila* embryo

To investigate dosage compensation in the early *Drosophila* embryo, we chose four X chromosome genes: *short gastrulation* (*sog*), *hindsight* (*hnt*), *giant* (*gt*), and *multiple edematous wings* (*mew*). These genes were chosen based on published time series RNA-seq data from male and female embryos, which found that *sog*, *gt*, and *hnt* are compensated in the early embryo, whereas *mew* is not effectively compensated.[29] To investigate the temporal dynamics of dosage-compensated transcription in the early *Drosophila* embryo, we utilized the MS2-MS2 coat protein (MCP) system to track nascent transcription at single-cell resolution in live embryos. CRISPR genome editing was used to introduce 24 copies of the MS2 loops into the large first intron of the endogenous *sog* and *mew* genes (Figure 1A). Insertion of the loops via CRISPR genome editing into the *sog* and *mew* genes did not alter expression of these genes and had little

effect on viability (Figures S1A–S1D). For *hnt* and *gt*, we utilized previously reported fly lines with 24 MS2 loops inserted into the 5′ and 3′ UTR sequences, respectively[30,31] (Figure 1A).

Females expressing MCP-GFP, His-RFP, and the endogenous X chromosome gene-MS2 were crossed to males carrying the same endogenous gene-MS2 insertion (Figure 1B). Live imaging of embryos from this cross revealed bright spots of fluorescence in nuclei corresponding to nascent transcription foci of the genes of interest, where the MCP-GFP is bound to the 24xMS2 loops within the nascent mRNA transcripts (Figure 1C). Some of the embryos imaged were female embryos with 2 active MS2 transcription sites; however, only movies of embryos carrying a single copy of the MS2-modified gene were analyzed. After imaging, each embryo was removed from the imaging dish, and genomic DNA was extracted and PCR amplified using X and Y chromosome-specific primers to sex the embryos (Figure 1B). For each gene, the nascent transcription site (TS) fluorescent signals were visible in nuclei within the expected expression domain; the fluorescence intensity of each TS is proportional to the number of transcribing RNA Pol II. A still from a movie from a *sogMS2* embryo is shown in Figure 1C.

Transcriptional activities of X chromosome genes in male and female embryos

For each gene and embryo sex, nascent TSs within the expression domain were imaged live in three replicate embryos. A representative movie is shown for each gene in male and female embryos in Videos S1, S2, S3, S4, S5, S6, S7, and S8. The timing of transcription in each embryo was related to developmental time using the onset of nc14 as a reference point. Each TS was assigned to a nucleus during nc14 to reveal the spatial expression domain (Figures 2A, 2D, 2G, and 2J) and tracked over time. For *sogMS2* embryos, we analyzed a consistent region by selecting nuclei within a fixed distance from the middle of the expression domain in either direction (Figure 2A). This excluded nuclei undergoing repression in the ventral region by Snail and those in the more dorsal region that have limiting activator.[32] Heatmaps of the mean fluorescence intensity traces from all nuclei analyzed across the 3 biological replicates for each gender show higher signals for some male nuclei (Figure 2B). Graphs of the *sogMS2* transcriptional activity show that the mean fluorescence intensity is lower for two out of the three female embryos analyzed (Figure 2C). The heatmaps also reveal that nuclei have a highly synchronous onset of transcription early in nc14 in both male and female embryos (Figure 2B).

In the *gtMS2* anterior expression domain (Figure 2D), there is synchronous onset of transcription in both sexes and a weak trend showing lower fluorescence in female embryos (Figures 2E and 2F). For *hntMS2*, we analyzed the posterior region of the embryo (Figure 2G) where there is higher transcription and the expression domain is at its broadest. Unlike *sogMS2* and *gtMS2*, *hntMS2* transcriptional traces have low synchronicity and show a broad range of onset times in both male and female embryos (Figure 2H). Two of the male *hntMS2* embryos have similar mean TS fluorescence intensities to two of the female embryos, although the other male and female embryos have higher and lower signals, respectively (Figure 2I). For both *gt* and *hnt*, some traces show fluctuating fluorescence signals, consistent with bursting.

For *mewMS2*, we imaged the dorsal side of the embryo and analyzed all cells of the expression domain (Figure 2J). Transcription onset is stochastic, and there are two peaks of transcription, with the second peak particularly evident in female embryos (Figure 2K). There is a weak trend of higher transcriptional activity in female embryos (Figure 2L). The live imaging data for the embryo replicates show some variation for each of the genes tested. This is likely biological variation, consistent with single-molecule fluorescence *in situ* hybridization (smFISH) quantitation of *gt* mRNAs revealing a spread of the mean total mRNA number/cell for different embryos of the same sex (Figures S1E–S1F). The similar range of mRNA numbers between the sexes (Figures S1F and S1G) is consistent with dosage compensation occurring.[29] Substantial fluctuations in mRNA numbers between embryos analyzed as a time series have also been reported.[33] Overall, these live imaging data suggest that there are some differences in the mean transcriptional activities between male and female embryos for the four X chromosome genes tested.

Dosage-compensated genes are not transcribed with a faster RNA Pol II elongation rate in males

Enhanced RNA Pol II elongation in males has been proposed to mediate dosage compensation.[18,19] Therefore, we next estimated RNA Pol II elongation rates from our MS2 data in male and female nc14 embryos. An autocorrelation function has been used previously to estimate RNA Pol II elongation time from live imaging data.[34–36] As the fluorescent signal at each TS is recorded at short time intervals that capture RNA Pol II transcribing the gene, the same MS2 mRNA with bound MCP-GFP will be present at multiple time points, resulting in successive fluorescence measurements being correlated. Therefore, the autocorrelation function decays linearly with a minimum value that corresponds to the dwell time of RNA Pol II/MS2 signal at the TS[34–36] (due to transcription termination in the case of *hnt*, or splicing for *sog* and *mew*).

A representative autocorrelation trace for *sog* in female embryos is shown in Figure 3A. The median dwell times for *sog*, *hnt*, and *mew* in each embryo tested are shown in Figure 3B; we did not include *gt* in this analysis, as the loops are located in the 3′ UTR and so the dwell time is extremely short. The data show that there is no significant difference between the dwell times for *hnt* in male and female embryos, whereas for *sog* and *mew*, there is a small but significant increase in elongation rate (shorter dwell time) in female embryos. Based on the gene length for *hnt*, the estimated dwell times suggest elongation rates of 2.7 and 2.8 kb/min in female and male embryos, respectively. These rates are consistent with the 1.4–3 kb/min range of elongation rates previously measured in the *Drosophila* embryo.[37–39] We have not converted the *sog* and *mew* dwell times to elongation rates, as we do not know how far RNA Pol II transcribes before the nascent mRNA is spliced given the variation in efficiency of co-transcriptional splicing in the embryo.[40]

Further analysis of the dwell times from nuclei located in different regions of the *sog*, *hnt*, and *mew* expression domains suggest that there is no spatial regulation of the RNA Pol II

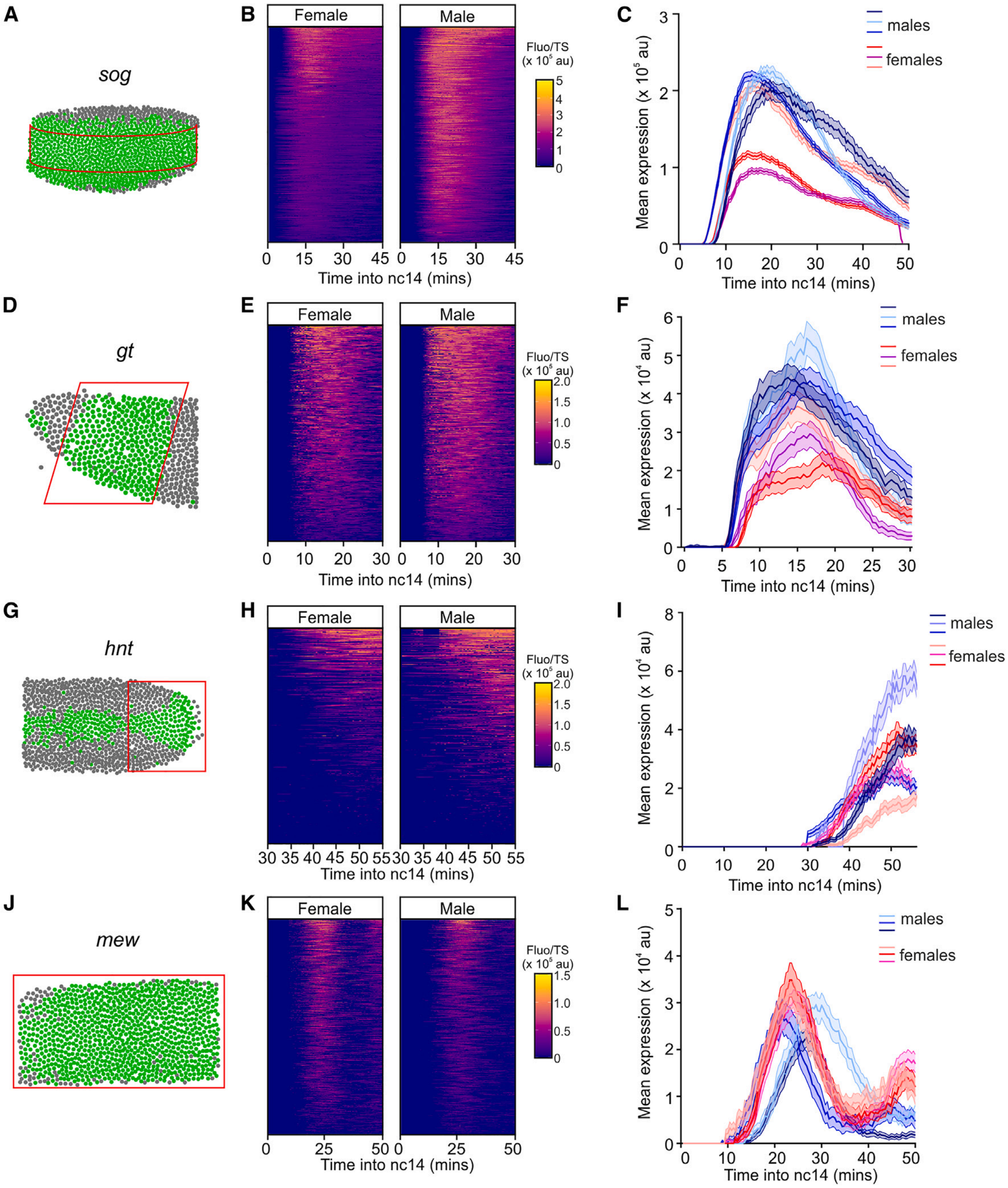

Figure 2. Transcriptional activities of X chromosome genes in male and female embryos

(A, D, G, and J) Schematics show a representative embryo with the expression domain for the indicated gene in green, based on active nuclei from the live imaging, and the area analyzed in the red box.

(B, E, H, and K) Heatmaps show the combined individual traces for transcriptionally active nuclei from all female and male embryos during nc14. Each row shows the transcriptional activity based on mean fluorescence intensity across developmental time in nc14. The fluorescence traces are ordered by total expression. All nuclear traces from 3 biological replicates of each sex are shown for each gene.

(C, F, I, and L) Graphs show the mean expression based on fluorescent signals for each of the 3 female and 3 male biological replicate embryos.

Mean ± 95% confidence intervals.

See also Figure S1.

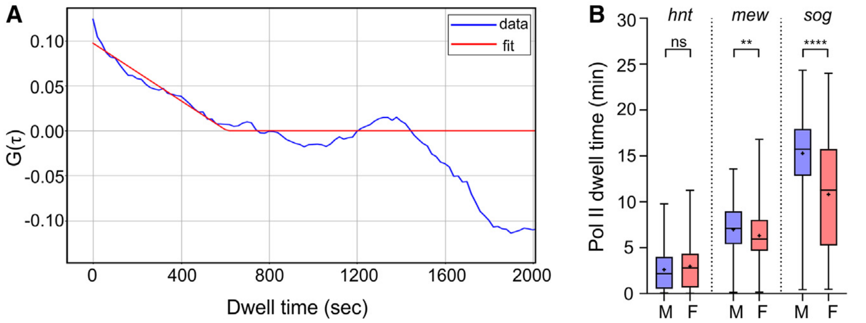

Figure 3. Estimation of RNA Pol II elongation rates in male and female embryos

(A) Graph shows the autocorrelation curve fit (red line) to a representative fluorescence trace (blue line) from a nucleus from a female *sogMS2* embryo. Fitting the function to the data gives the dwell time.
(B) Boxplot shows the median dwell time based on the data from nuclei across three biological replicate embryos for *hnt*, *mew*, and *sog*. n = 533 (*sog* female) and 516 (*sog* male), 247 (*mew* female) and 202 (*mew* male), and 194 (*hnt* female) and 187 (*hnt* male). Boxes show 25th to 75th percentile, and whiskers show range; mean is indicated with a plus (+) symbol; Welch's t test $^{**}p < 0.01$, $^{****}p < 0.0001$; ns, not significant.
See also Figure S2.

elongation rates (Figures S2A–S2C). However, due to limitations with this analysis, we excluded nuclei with sparse fluorescent traces (see STAR Methods) that are typically on the edge(s) of the expression domain, so we cannot rule out changes in the elongation rate in these regions. Nonetheless, as in the global analysis of RNA Pol II elongation rate, we observe no significant difference for *hnt* between male and female embryos, whereas faster elongation rates were estimated for *sog* and *mew* in female nuclei (Figures S2A–S2C). Together, these data do not support faster RNA Pol II transcription in males for the dosage-compensated genes tested.

sog hypertranscription in males is due to a higher RNA Pol II initiation rate

As there was no significant increase in male RNA Pol II elongation rates, we investigated transcriptional regulation in more detail by inferring the parameters associated with transcriptional bursting. During transcriptional bursting, when the promoter switches from the on state to the off state, fluorescence persists due to RNA Pol II molecules transcribing the gene body. Therefore, we used a memory-adjusted hidden Markov model to infer the rates and bursting parameters from the MS2 transcriptional traces in male and female embryos.[35,41] The model is based on a two state model of transcriptional bursting in which the promoter switches between on and off states with rates k_{on} and k_{off} and initiates transcription with a rate k_{ini} when the promoter is in the on state. k_{on} and k_{ini} dictate burst frequency and amplitude, respectively, whereas burst duration is equivalent to $1/k_{off}$ (Figure 4A). Promoter occupancy, based on k_{on} and k_{off}, is the fraction of time the promoter is in an active state[42] (Figure 4A).

We used the live imaging data from nuclei in the analyzed regions of the expression domain to infer burst parameters at single-cell resolution in nc14 for each embryo, as described previously.[31,41] We then pooled the data for the 3 embryos of each sex and calculated the mean parameter from nuclei divided into single-cell-wide bins moving across the expression domain at nc14. For the analysis of *sog* transcription, the single-cell bins are positioned along the dorsal-ventral (DV) axis and move dorsally (Figure 4B), as *sog* is activated by the dorsal gradient.[32] This analysis reveals that mean total expression is ~1.5 fold higher in each row of nuclei in male embryos relative to females (Figure 4C), consistent with the whole-embryo trends shown in Figure 2. This magnitude of effect is in the expected range for dosage compensation, which, if complete, is predicted to increase transcription 2-fold.

RNA Pol II initiation rate is ~1.8 fold higher for *sog* in each spatial bin in male embryos (Figure 4D), whereas promoter occupancy is slightly higher in female embryos, particularly in ventral nuclei (low-numbered bins) (Figure 4E). This increase in occupancy in females in ventral nuclei is due to a small increase in k_{on} and a decrease in k_{off} (Figures 4F and 4G). These results suggest that the higher RNA Pol II initiation rate on the *sog* transcription output in males is negated to some extent by the lower promoter occupancy, as the promoter spends less time in the on state in male nuclei.

The total transcription output depends on occupancy, loading rate, and the transcription time window.[35,38] We therefore calculated the time window of *sog* transcription in each nucleus by using the fluorescence data to calculate the difference between the time when transcription is first detected (t_{on}) and then turns off (t_{off}). This analysis reveals that male and female embryos have a similar time window in most nuclei, with only a very minor extension of the time window (up to 3 min longer) in some of the male embryo bins (Figures 4H, S3A, and S3B). For some nuclei, *sog* transcription is still detectable when the embryo starts to gastrulate, but the cell movements prevent continued tracking of the TSs. Therefore, in this analysis, we have used the end of the imaging period as an estimate for t_{off} for these nuclei (see STAR Methods). However, we were able to calculate a t_{off} value earlier than the end of the imaging time for more nuclear traces from female embryos than males (Figure S3C), suggesting that males have a longer time window for some nuclei. By multiplying the RNA Pol II initiation rate, the promoter occupancy, and the time window, we estimate a higher total transcription output in male embryos than females, with similar relative outputs to those observed based on the mean total fluorescent signal (Figure S3D). Together, these data suggest that dosage compensation of *sog* is mediated by a higher RNA Pol II initiation rate in males, with the magnitude of the increase dampened by a lower promoter occupancy.

Dosage-compensated genes have elevated transcription burst amplitude in male embryos

Next, we investigated the bursting parameters for *gt*. As *gt* is activated by the Bicoid (Bcd) gradient,[43] we used spatial bins moving across the embryo from the anterior to posterior of the

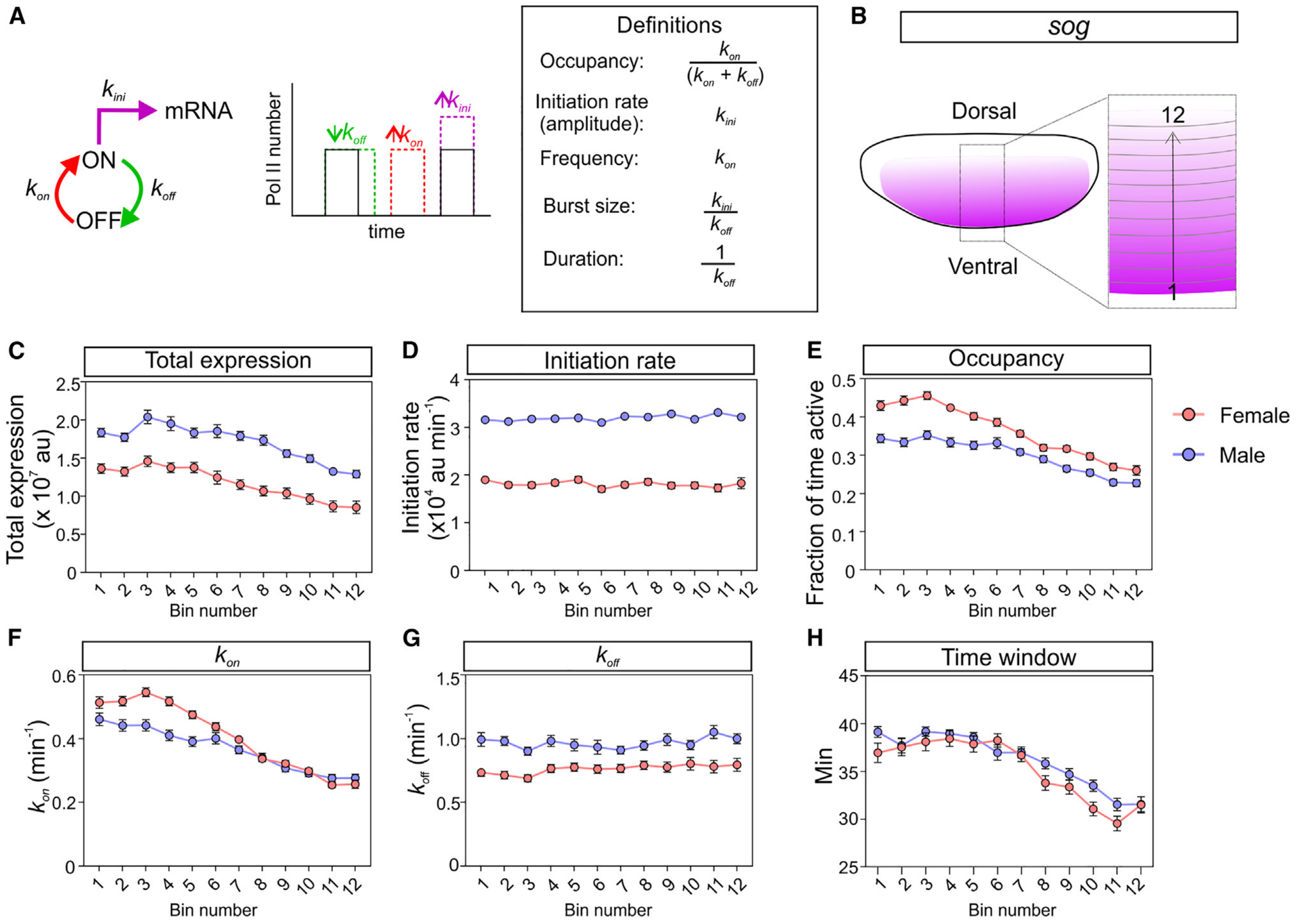

Figure 4. ***sog* hypertranscription in males is due to a higher amplitude of transcriptional bursts**

(A) Overview of the two-state model of transcriptional bursting, showing k_{on}, k_{off}, and k_{ini} and parameter definitions. The effect of changes in these rates on bursting is shown on the graph.

(B) Cartoon shows a schematic of the *sog* expression domain (ventrolateral view) with the single-cell bins numbered from the ventral side of the expression domain.

(C) Graph shows the mean total expression from nuclei in each single-cell bin. Each male and female data point shows the data from nuclei pooled from 3 biological replicates.

(D–H) Graphs show the binned single-cell transcriptional parameters inferred from the *sogMS2* transcriptional traces from male (blue) and female (red) embryos: (D) RNA Pol II initiation rate, (E) promoter occupancy, (F) k_{on}, (G) k_{off}, and (H) transcription time window.

Mean ± SEM, n = 1,076 nuclei (female) and 1,081 nuclei (male) pooled from 3 biological replicates for each sex.

See also Figure S3.

expression domain. Mean total expression shows a drop near the center of the expression domain, consistent with the refinement of the broad anterior band of expression into 2 stripes.[43] Analysis of bursting parameters reveals that there is a small (~1.4 fold) increase in total transcription in some, but not all, male nuclei that are transcribing *gtMS2* (Figure 5A). Promoter occupancy is similar for both sexes; although k_{on} is higher in female nuclei in some of the bins, the higher k_{off} in females results in a shorter burst duration (Figure 5A). RNA Pol II initiation rate shows a small (~1.4 fold) increase across the majority of the bins in male embryos (Figure 5A). The transcription time window is longer—up to 8 min—in many of the male nuclei bins due to later a t_{off} (Figures S3E–S3G). Similar to *sogMS2*, we saw that more female than male nuclei completed transcription within the imaging time period, consistent with males having an extended transcriptional window (Figure S3H). Multiplying the promoter occupancy, the RNA Pol II initiation rate, and the time window predicts the spatial trends of mean total expression across the expression domain in male and female *gtMS2* embryos (Figure S3I). In summary, small increases in RNA Pol II initiation rate and, for some nuclei, the time window of transcription lead to modest increases in *gt* expression in male embryos.

As *hnt* transcription is activated by Dpp signaling,[44] the single-cell parameters were grouped in DV bins moving across the dorsal midline (Figure 5B). As *hnt* transcription starts late in nc14, and as peak expression is only reached late in the imaging period (Figure 2I), we were unable to accurately estimate the transcription time window. Mean expression is ~1.5-fold higher in male

CellPress
OPEN ACCESS

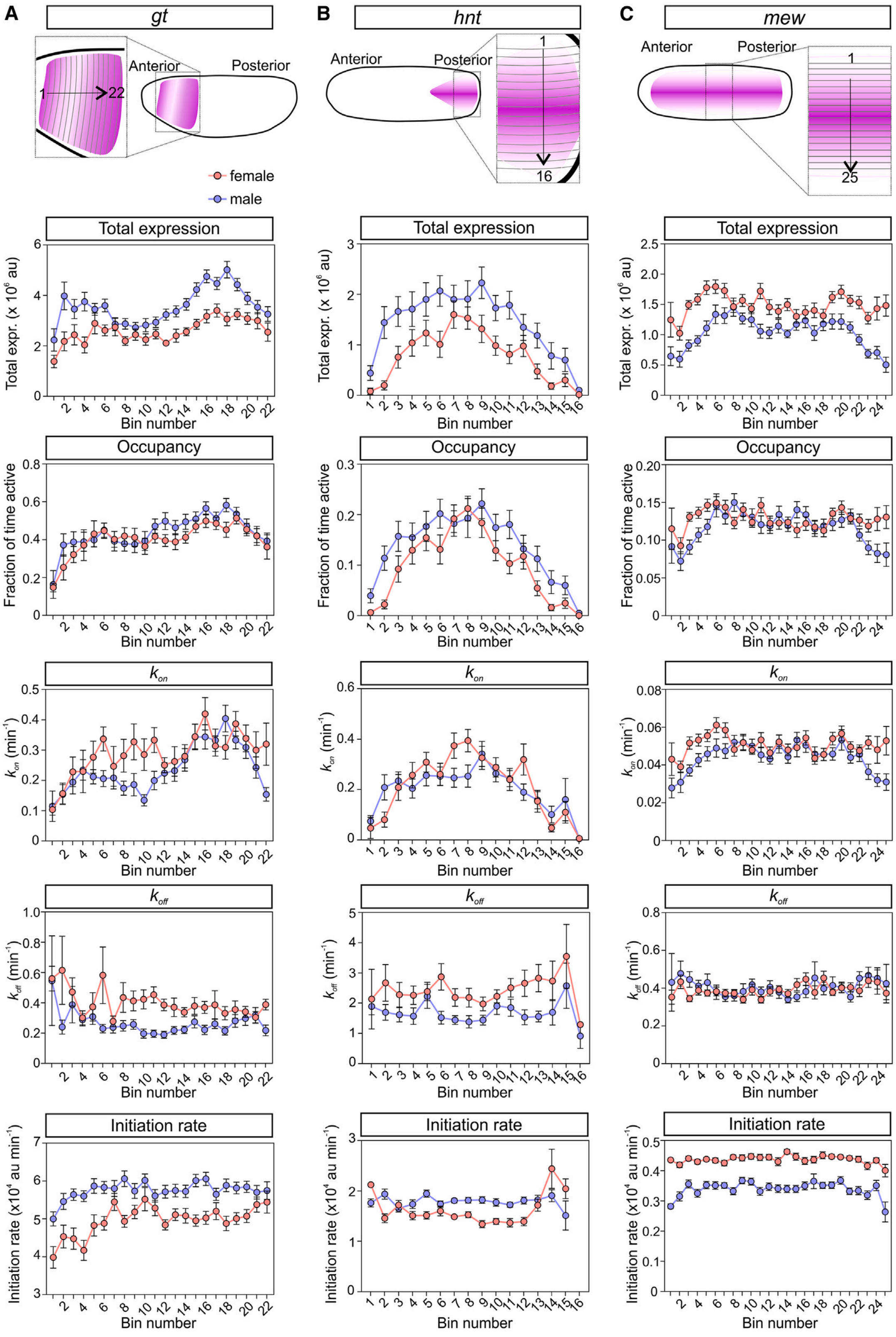

(legend on next page)

embryos due to small increases in both the promoter occupancy (based on lower k_{off}) and RNA Pol II initiation rate (Figure 5B). The *hnt* parameters inferred here in the posterior of the embryo are similar to those reported previously for *hnt* transcription in nuclei in the center of the expression domain.[31]

For *mew*, we analyzed the single-cell parameters in DV spatial bins moving across the dorsal midline (Figure 5C). This analysis reveals that, in contrast to the other X chromosome genes, mean expression is slightly higher in female embryos. This appears to be driven by an increased RNA Pol II initiation rate, which is higher in female nuclei in all of the bins. In contrast, promoter occupancy and k_{on} only show small increases at each end of the expression domain in female nuclei, whereas k_{off} is unchanged (Figure 5C). Together, the single-cell bursting parameter data suggest that *sog*, *gt*, and *hnt* are dosage compensated at the transcriptional level. Promoter occupancy is modulated to different degrees for transcription of these genes, and the time window of active transcription is increased for *gt* and, to a lesser extent, for *sog*. However, all 3 genes show increases in the RNA Pol II initiation rate in males. In contrast, the RNA Pol II initiation rate is higher in female nuclei for *mew*, which is not dosage compensated,[29] and even shows a higher transcriptional output in females.

Burst frequency and promoter occupancy control transcriptional changes across the expression domain

Having investigated how bursting parameters change for transcription of the X chromosome genes between male and female embryos, we next used the single-cell parameters to determine which parameter underpins the transcriptional changes observed spatially across each expression domain. To this end, for each embryo, we visualized mean expression and the individual parameters spatially as both heatmaps and graphs with each point representing the data from a single nucleus in the expression domain (Figures 6A and 6B). In addition, we calculated the correlation between mean expression and each parameter, as described previously.[31] Analysis of the data for *sog* transcription reveals that mean expression declines in nuclei positioned more dorsally in the expression domain (Figures 6A and 6B), consistent with reduced levels of the dorsal activator.[32] Testing the correlation between mean expression and the different parameters reveals that promoter occupancy is most correlated, whereas there is little correlation between the mean expression profile across the expression domain and the RNA Pol II initiation rate, which is largely unchanged (Figure 6B; correlations for the other biological replicate embryos are shown in Figure S4A). Consistent with occupancy being highly correlated, both k_{on} and k_{off} are the parameters that are next most strongly correlated with mean expression (Figures 6B and S4A).

Analysis of the *gt*, *hnt*, and *mew* single-cell parameters across the expression domain and their correlation with mean expression also reveals that promoter occupancy is most highly correlated, to the extent that it can accurately predict the mean expression profile in each embryo (Figures 6C, 6D, and S4B–S4G). For all genes, k_{on} is strongly correlated, with k_{off} for *mew* also showing a strong correlation with mean expression (Figures 6C, 6D, and S4B–S4G). Together, these data show how different bursting parameters are modulated to alter the transcription output in embryos in distinct ways. Our data suggest that nuclei respond to activator concentration through changes in promoter occupancy and burst frequency (k_{on}), whereas sex-specific modulation of the RNA Pol II initiation rate further defines the transcription output.

DISCUSSION

Here, we use live imaging to analyze the transcriptional burst kinetics for four X chromosome genes at single-cell resolution. For the 3 genes previously shown to be dosage compensated,[29] we detect significantly higher mean transcriptional activity in all (*sog*) or many (*gt* and *hnt*) of the single-cell bins across the expression domain in male embryos. In contrast, *mew*, which was chosen as a negative control since it is not compensated,[29] shows higher transcription in female embryos. The reason for this difference in *mew* transcription in female embryos is currently unclear, but it is possible that X chromosome genes, which are not compensated, are under transcribed in males. The one bursting parameter in common with all these sex-specific changes in transcription output is the RNA Pol II initiation rate, suggesting that control of burst amplitude underpins the transcriptional changes associated with dosage compensation. We find little change in the RNA Pol II initiation rate across the expression domain in response to changes in activator concentration, but instead, it appears to be differentially tuned depending on embryo sex.

It has been shown for transcription of gap genes in *Drosophila* nc13 embryos that the initiation rate is constant for the different gap genes tested and at all positions across the expression domains.[42] While k_{on} and k_{off} have been reported to be regulated in response to differing concentrations of many transcription factors and cell signals,[45,46] examples where the RNA Pol II initiation rate is modulated are rarer. One example is during the refinement of the seven *even-skipped* stripes in the *Drosophila* embryo. Following the onset of their transcription, both k_{on} and k_{ini} are upregulated in the center of each stripe as their expression domains narrow.[47,48] Modulation of the RNA Pol II initiation rate also occurs at a global level to scale transcription to changes in cell size in *Schizosaccharomyces pombe*. In this model, genes

Figure 5. Differences in burst amplitude are associated with sex-specific changes in transcription
(A) Cartoon shows the *gt* expression domain with the numbered single-cell bins used in the analysis. The graphs show the mean total expression, promoter occupancy, k_{on}, k_{off}, and RNA Pol II initiation rate in each bin from nuclei from male (blue) and female (red) embryos. The data and single-cell parameters from nuclei in 3 biological replicate embryos of each sex were pooled and reported in the indicated bins.
(B and C) As in (A), but the data are shown for *hnt* (B) and *mew* (C).
Mean ± SEM, n = 437 (*gt* female) and 675 nuclei (*gt* male), 387 (*hnt* female) and 420 (*hnt* male), and n = 1,067 (*mew* female) and 894 (*mew* male) pooled from 3 biological replicates for each sex.
See also Figure S3.

CellPress
OPEN ACCESS

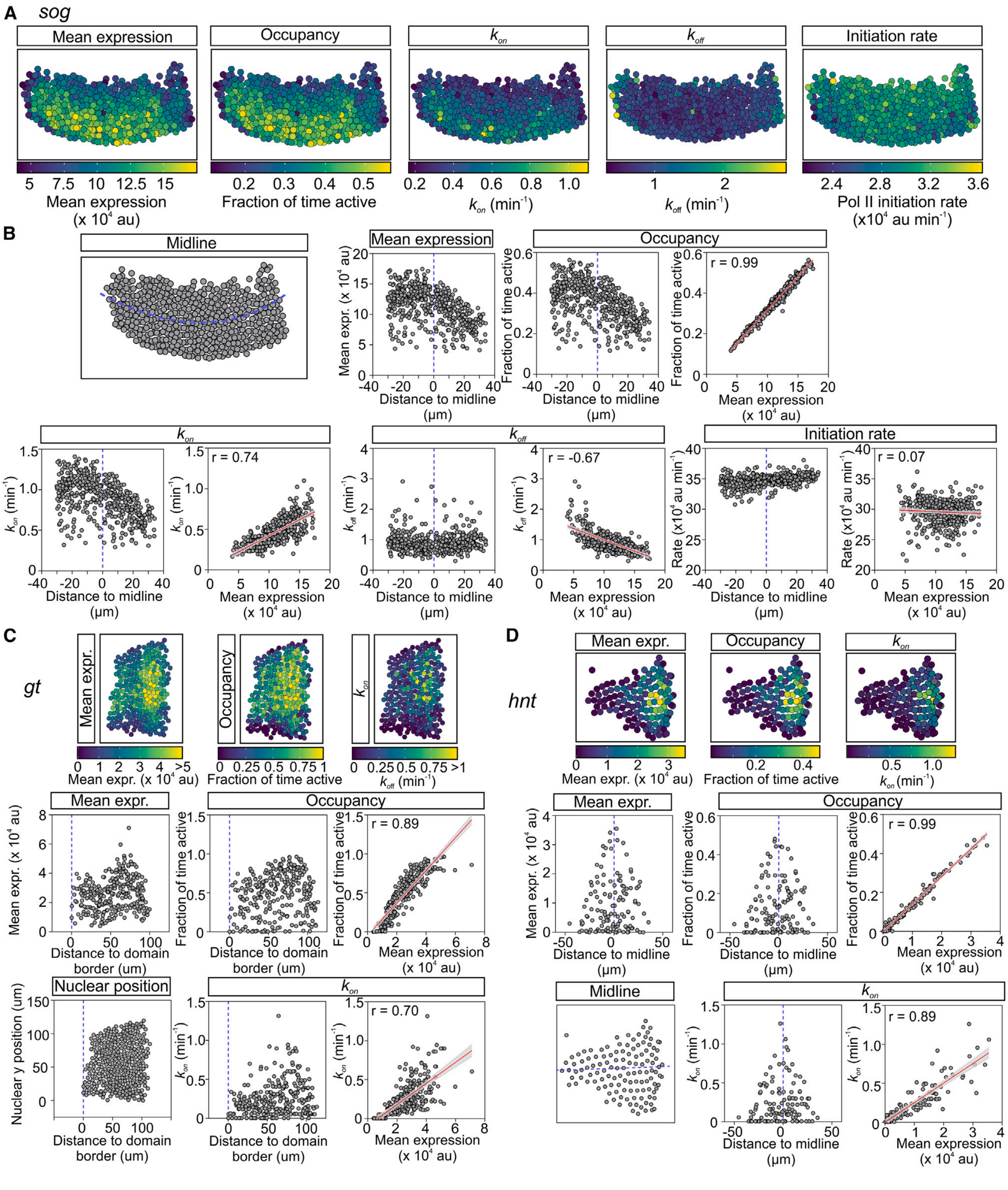

Figure 6. Changes in burst frequency and promoter occupancy underpin the transcriptional changes across the expression domain

(A) Heatmaps show the *sogMS2* expression domain with nuclei colored as in the associated key for mean expression, promoter occupancy, k_{on}, k_{off}, and RNA Pol II initiation rate.

(B) Schematic shows the *sogMS2* expression domain with its midline marked in blue. Mean expression for each nucleus in the embryo is plotted based on its position from the expression domain midline with ventral (negative) and dorsal (positive) distances. For promoter occupancy, k_{on}, k_{off}, and RNA Pol II initiation rate,

(legend continued on next page)

compete for limiting RNA Pol II, and the amount of chromatin-bound RNA Pol II increases with cell size.[49] Therefore, as we find different RNA Pol II initiation rates in male and female embryos for the four X chromosome genes studied, we speculate that regulation of the RNA Pol II initiation rate may be a strategy primarily deployed by the cell to modulate transcriptional responses at a whole-chromosome or -transcriptome level.

Recent analysis of dosage compensation in the embryo has revealed that there is a maternal MSL subcomplex composed of MSL1, MSL3, and MOF, with the latter subunit acetylating H4K16 on all chromosomes in ovaries and pre-blastoderm embryos. This active mark is enriched at promoters prior to zygotic genome activation and increases nucleosome accessibility, priming genes for subsequent activation.[20] The *msl-2* mRNA is detectable in both sexes of embryo at nc13 and continues to accumulate until the mRNA level declines in females at mid-nc14 but increases further in male embryos.[29] MSL-2 protein was detected as diffuse X chromosome staining in male nc14 embryos, consistent with the canonical MSL complex becoming active at this stage. Moreover, knockdown of maternal MOF results in a reduction of transcription from all chromosomes at nc14, but there is a stronger downregulation of transcription from genes proximal to HASs in males that is not observed in female embryos.[20] Given this timing, the canonical MSL complex could mediate the increased transcription we observe in nc14 for the dosage-compensated genes. However, there is also the inverse dosage model of dosage compensation in which the MSL complex has no direct role in X chromosome transcription, based in part on considerations around normalization and interpretation of genomic data from MSL loss-of-function studies.[15] We note that analysis of live transcription of X chromosome and autosome genes in MSL mutants may help resolve whether or not the MSL complex acts directly on X chromosome transcription.

Non-canonical dosage compensation has also been proposed in the early *Drosophila* embryo,[29,50–52] which is sex-lethal (Sxl) dependent but MSL independent.[50,51] Based on the observation that multiple Sxl binding sites are more prevalent in the 3′ UTR of mRNAs transcribed from the X chromosome than the autosomes,[52,53] a model was suggested whereby Sxl destabilizes or represses translation of mRNAs in female embryos.[52] Additionally, miRNAs have been implicated in genomic balance and dosage compensation.[54] It is possible that transcriptional and post-transcriptional mechanisms function together to equalize mRNA levels between males and females, as the transcriptional changes we observe (~1.5-fold) are lower than the complete dosage compensation suggested by RNA-seq[29] and our *gt* smFISH data. There is a precedent for this, as increased stability of mRNAs from the X chromosome compared with the autosomes has been proposed to function with hypertranscription in mammalian cells to allow dosage compensation.[55] Recently, zygotic mRNA half-lives have been estimated in female *Drosophila* embryos.[56] This approach could be used to determine whether dosage-compensated X chromosome mRNAs are more stable in male embryos.

In terms of an initiation-[24–27] vs. elongation-based[18,19] model of transcriptional hyperactivation in males, the telegraph model cannot distinguish between the changes in the RNA Pol II initiation rate being due to effects on RNA Pol II recruitment or pause release. However, recent kinetic data from *Drosophila* and human tissue culture cells suggest that paused RNA Pol II does not represent an essential state in between the off and permissive on states. Instead, pausing is a rare alternative off state, which cannot be captured as a distinct off state for all promoters but is relatively long lived when it exists.[57,58] Based on this, we favor a recruitment-based model, although further studies are required to address this. As well as studying additional X chromosome genes, models that incorporate an additional pausing state[57,58] could be used. Our data do not support faster RNA Pol II elongation on the bodies of the genes tested in males, as suggested previously,[18,19] with the opposite effect observed for *sog* transcription. However, as we only performed this analysis for *sog* and *hnt* as examples of compensated genes, it is possible that other male X chromosome genes are transcribed at a faster speed than in female embryos.

In addition to an altered RNA Pol II initiation rate, we also detect a small increase in the time window of active transcription for *sog* and *gt* in male embryos. This is due to a later t_{off}, with transcription still active at the end of our analysis period for more male than female nuclei. Modulation of the transcriptional time window is critical for generating the *eve* stripe 2 pattern, with control of the window arising from different off times.[35] Tethering elements have recently been described that mediate long-range enhancer-promoter interactions and associations between the promoters of paralogous genes that allow coupling of transcription dynamics.[59,60] Loss of tethering elements alters the timing of activation and bursting dynamics.[60] CLAMP and GAF, which both recruit the MSL complex,[61] bind tethering elements,[60] raising the possibility that CLAMP/GAF on the male X may influence the transcription time window and bursting. Alternatively, the MOF-deposited H4K16ac and action of the canonical MSL complex at nc14[20] may facilitate nucleosome accessibility on the male X for longer.

Recently the *roX* RNAs and MSL2, which has an intrinsically disordered C-terminal domain, have been found to form a stable X chromosome territory in males, which has many features of a phase-separated condensate.[14] We suggest that the male X chromosome territory concentrates RNA Pol II, increasing the number of molecules available for transcription of X chromosome genes, thereby elevating the RNA Pol II initiation rate and potentially the time of active transcription. X chromosome genes that are not compensated, such as *mew*, may be excluded from

the left graph shows the value for each nucleus plotted against expression domain position. The right graph shows the correlation (red line with 95% CI) between the indicated parameter and mean expression based on the data from each individual nucleus in the expression domain for a single embryo.

(C and D) As in (A) and (B) except the data are for *gtMS2* and *hntMS2* and only the graphs for mean expression, promoter occupancy, and k_{on} are shown. Data for the other *gtMS2* and *hntMS2* parameters and for *mew* are shown in Figure S4. Data represent n = 475 (*sog*), 280 (*gt*), and 122 nuclei (*hnt*) from one representative embryo. Full data including all biological replicates for each gene can be found in Figure S4.

See also Figure S4.

or have an unfavorable position in the male X territory. However, further work is required to test this model, and we note that RNA Pol II exclusion from the inactive X chromosome during dosage compensation in mammals does not depend on biophysical compartmentalisation.[62]

We also investigated how bursting parameters are regulated in space across the expression domain. We find higher mean transcription in areas where there is increased activator concentration, e.g., in *sog* ventral nuclei or *gt* anterior nuclei.[32,43] Our data reveal that regulation of promoter occupancy, the proportion of time the promoter is active, underpins the observed transcriptional changes across the expression domain of X chromosome genes. This parameter is also tuned to establish the transcription profiles of the *Drosophila* gap genes at nc13[42] and nc14[30] and the response of target genes to different BMP signaling levels.[31] Our data suggest that occupancy is primarily modulated by changes in burst frequency (k_{on}) in response to activator concentration. This is consistent with other reports of transcription factor concentration regulating burst frequency[45] due to a reduction in the search time for the enhancer.[36] k_{off} also negatively correlates with mean expression of *sog* and *mew* in particular. Burst duration ($1/k_{off}$) depends on the transcription factor dwell time,[63] suggesting that dwell time differs positionally across the expression domain, potentially due to cooperative interactions with another, more localized transcription factor. Regulation of burst duration has been proposed to mediate the transcriptional response to different levels of Notch signaling.[64,65] Overall, our data suggest that the transcription output of X chromosome genes depends on two tiers of inputs. Parameters such as burst frequency are locally tuned in nuclei within the expression domain in response to varying transcription factor inputs, whereas burst amplitude is set globally by the sex of the embryo.

Limitations of the study

While previous genomics-based studies of dosage compensation have allowed all active X chromosome genes to be studied,[18,19,24] we have focused on transcription of only 4 X chromosome genes due to the low-throughput nature of MS2 imaging. As complete dosage compensation of male X chromosome genes would result in a maximum 2-fold effect at the transcriptional level, in the context of biological variation analysis of the live imaging data is not straightforward. By binning our data spatially and pooling nuclei across biological replicates of the same sex, we present evidence that burst amplitude modulation contributes to the sex-specific expression changes observed for all of the genes we studied. However, as discussed above, the two-state model cannot distinguish between a higher initiation rate due to increased RNA Pol II recruitment or enhanced pause release.

As we detected higher burst amplitude for all 3 compensated genes, we speculate that this will be a general mechanism for hypertranscription of male X chromosome genes, but further work is needed to address this. One option for inferring burst parameters for multiple X chromosome genes would be to exploit an approach for burst inference based on the two-state model that was described for allele-specific single-cell RNA-seq (scRNA-seq)[66] and that has recently been used with *Drosophila* scRNA-seq data.[67] This approach can estimate k_{on} and burst size (k_{ini}/k_{off}), although estimation of the individual k_{ini} and k_{off} parameters is less reliable.[66] However, as noted,[68] the sparsity of reads in existing scRNA-seq data from the *Drosophila* embryo[68,69] makes sexing the nuclei difficult. In addition, without allele-specific scRNA-seq data, the model requires an additional parameter, the frequency with which the bursting from the two alleles in female nuclei is coordinated. As the frequency of co-bursting is not trivial to estimate and likely changes across expression domains, inferring allele-specific burst parameters for multiple X chromosome genes from scRNA-seq data is currently challenging.

STAR★METHODS

Detailed methods are provided in the online version of this paper and include the following:

- KEY RESOURCES TABLE
- RESOURCE AVAILABILITY
  - Lead contact
  - Materials availability
  - Data and code availability
- EXPERIMENTAL MODEL AND SUBJECT DETAILS
  - Experimental animals and crosses
- METHOD DETAILS
  - CRISPR of 24X MS2 loops into endogenous loci
  - Single molecule fluorescent *in situ* hybridisation
  - PCR assay to identify embryo sex
  - Viability assay
  - Confocal microscopy of fixed embryos
  - Live imaging microscopy
  - Image deconvolution
- QUANTIFICATION AND STATISTICAL ANALYSIS
  - Live and fixed embryo image analysis
  - Autocorrelation estimation of elongation time
  - Modeling transcriptional parameters

SUPPLEMENTAL INFORMATION

Supplemental information can be found online at https://doi.org/10.1016/j.celrep.2023.112382.

ACKNOWLEDGMENTS

We thank Caroline Hoppe, Jennifer Love, Catherine Sutcliffe, and Sophie Frampton for comments on the manuscript; Takashi Fukaya for the *gtMS2* flies; Catherine Sutcliffe for technical help; the Bloomington Drosophila Stock Center for flies; the Cambridge Fly Facility for microinjections; and the University of Manchester Bioimaging and Fly facilities for support. This project was supported by a Wellcome Trust Investigator award to H.L.A. and M.R. (204832/Z/16/Z and 204832/B/16/Z).

AUTHOR CONTRIBUTIONS

Conceptualization, L.F.B., M.R., and H.L.A.; investigation, L.F.B. and H.Z.; writing, L.F.B. and H.L.A.; supervision, M.R. and H.L.A.; funding acquisition, M.R. and H.L.A.

DECLARATION OF INTERESTS

The authors declare no competing interests.

INCLUSION AND DIVERSITY

We support inclusive, diverse, and equitable conduct of research.

Received: July 25, 2022
Revised: February 3, 2023
Accepted: March 27, 2023
Published: April 14, 2023

REFERENCES

1. Muller, H.J. (1932). Further studies on the nature and causes of gene mutations. Proc. Sixth Int. Congr. Genet. *1*, 213–255.
2. Belote, J.M., and Lucchesi, J.C. (1980). Control of X chromosome transcription by the maleless gene in Drosophila. Nature *285*, 573–575. https://doi.org/10.1038/285573a0.
3. Lakhotia, S.C., and Mukherjee, A.S. (1969). Chromosomal basis of dosage compensation in *Drosophila* : I. Cellular autonomy of hyperactivity of the male *X* -chromosome in salivary glands and sex differentiation. Genet. Res. *14*, 137–150. https://doi.org/10.1017/S001667230000197X.
4. Mukherjee, A.S., and Beermann, W. (1965). Synthesis of ribonucleic acid by the X-chromosomes of Drosophila melanogaster and the problem of dosage compensation. Nature *207*, 785–786. https://doi.org/10.1038/207785a0.
5. Kuroda, M.I., Hilfiker, A., and Lucchesi, J.C. (2016). Dosage compensation in drosophila–a model for the coordinate regulation of transcription. Genetics *204*, 435–450. https://doi.org/10.1534/genetics.115.185108.
6. Samata, M., and Akhtar, A. (2018). Dosage compensation of the X chromosome: a complex epigenetic assignment involving chromatin regulators and long noncoding RNAs. Annu. Rev. Biochem. *87*, 323–350. https://doi.org/10.1146/annurev-biochem-062917-011816.
7. Alekseyenko, A.A., Peng, S., Larschan, E., Gorchakov, A.A., Lee, O.-K., Kharchenko, P., McGrath, S.D., Wang, C.I., Mardis, E.R., Park, P.J., and Kuroda, M.I. (2008). A sequence motif within chromatin entry sites directs MSL establishment on the Drosophila X chromosome. Cell *134*, 599–609. https://doi.org/10.1016/j.cell.2008.06.033.
8. Straub, T., Grimaud, C., Gilfillan, G.D., Mitterweger, A., and Becker, P.B. (2008). The chromosomal high-affinity binding sites for the Drosophila dosage compensation complex. PLoS Genet. *4*, e1000302. https://doi.org/10.1371/journal.pgen.1000302.
9. Kelley, R.L., Meller, V.H., Gordadze, P.R., Roman, G., Davis, R.L., and Kuroda, M.I. (1999). Epigenetic spreading of the Drosophila dosage compensation complex from roX RNA genes into flanking chromatin. Cell *98*, 513–522. https://doi.org/10.1016/S0092-8674(00)81979-0.
10. Oh, H., Park, Y., and Kuroda, M.I. (2003). Local spreading of MSL complexes from roX genes on the Drosophila X chromosome. Genes Dev. *17*, 1334–1339. https://doi.org/10.1101/gad.1082003.
11. Park, Y., Kelley, R.L., Oh, H., Kuroda, M.I., and Meller, V.H. (2002). Extent of chromatin spreading determined by *roX* RNA recruitment of MSL proteins. Science *298*, 1620–1623. https://doi.org/10.1126/science.1076686.
12. Soruco, M.M.L., Chery, J., Bishop, E.P., Siggers, T., Tolstorukov, M.Y., Leydon, A.R., Sugden, A.U., Goebel, K., Feng, J., Xia, P., et al. (2013). The CLAMP protein links the MSL complex to the X chromosome during Drosophila dosage compensation. Genes Dev. *27*, 1551–1556. https://doi.org/10.1101/gad.214585.113.
13. Rieder, L.E., Jordan, W.T., and Larschan, E.N. (2019). Targeting of the dosage-compensated male X-chromosome during early Drosophila development. Cell Rep. *29*, 4268–4275.e2. https://doi.org/10.1016/j.celrep.2019.11.095.
14. Valsecchi, C.I.K., Basilicata, M.F., Georgiev, P., Gaub, A., Seyfferth, J., Kulkarni, T., Panhale, A., Semplicio, G., Manjunath, V., Holz, H., et al. (2021). RNA nucleation by MSL2 induces selective X chromosome compartmentalization. Nature *589*, 137–142. https://doi.org/10.1038/s41586-020-2935-z.
15. Birchler, J.A. (2016). Parallel universes for models of X chromosome dosage compensation in *Drosophila*: a review. Cytogenet. Genome Res. *148*, 52–67. https://doi.org/10.1159/000445924.
16. Sun, L., Fernandez, H.R., Donohue, R.C., Li, J., Cheng, J., and Birchler, J.A. (2013). Male-specific lethal complex in *Drosophila* counteracts histone acetylation and does not mediate dosage compensation. Proc. Natl. Acad. Sci. USA *110*, E808–E817. https://doi.org/10.1073/pnas.1222542110.
17. Veitia, R.A., Bottani, S., and Birchler, J.A. (2008). Cellular reactions to gene dosage imbalance: genomic, transcriptomic and proteomic effects. Trends Genet. *24*, 390–397. https://doi.org/10.1016/j.tig.2008.05.005.
18. Ferrari, F., Plachetka, A., Alekseyenko, A.A., Jung, Y.L., Ozsolak, F., Kharchenko, P.V., Park, P.J., and Kuroda, M.I. (2013). Jump start and gain" model for dosage compensation in Drosophila based on direct sequencing of nascent transcripts. Cell Rep. *5*, 629–636. https://doi.org/10.1016/j.celrep.2013.09.037.
19. Larschan, E., Bishop, E.P., Kharchenko, P.V., Core, L.J., Lis, J.T., Park, P.J., and Kuroda, M.I. (2011). X chromosome dosage compensation via enhanced transcriptional elongation in Drosophila. Nature *471*, 115–118. https://doi.org/10.1038/nature09757.
20. Samata, M., Alexiadis, A., Richard, G., Georgiev, P., Nuebler, J., Kulkarni, T., Renschler, G., Basilicata, M.F., Zenk, F.L., Shvedunova, M., et al. (2020). Intergenerationally maintained histone H4 lysine 16 acetylation is instructive for future gene activation. Cell *182*, 127–144.e23. https://doi.org/10.1016/j.cell.2020.05.026.
21. Kind, J., Vaquerizas, J.M., Gebhardt, P., Gentzel, M., Luscombe, N.M., Bertone, P., and Akhtar, A. (2008). Genome-wide analysis reveals MOF as a key regulator of dosage compensation and gene expression in Drosophila. Cell *133*, 813–828. https://doi.org/10.1016/j.cell.2008.04.036.
22. Gelbart, M.E., Larschan, E., Peng, S., Park, P.J., and Kuroda, M.I. (2009). Drosophila MSL complex globally acetylates H4K16 on the male X chromosome for dosage compensation. Nat. Struct. Mol. Biol. *16*, 825–832. https://doi.org/10.1038/nsmb.1644.
23. Smith, E.R., Pannuti, A., Gu, W., Steurnagel, A., Cook, R.G., Allis, C.D., and Lucchesi, J.C. (2000). The *Drosophila* MSL complex acetylates histone H4 at lysine 16, a chromatin modification linked to dosage compensation. Mol. Cell Biol. *20*, 312–318. https://doi.org/10.1128/MCB.20.1.312-318.2000.
24. Conrad, T., Cavalli, F.M.G., Vaquerizas, J.M., Luscombe, N.M., and Akhtar, A. (2012). Drosophila dosage compensation involves enhanced Pol II recruitment to male X-linked promoters. Science *337*, 742–746. https://doi.org/10.1126/science.1221428.
25. Ferrari, F., Jung, Y.L., Kharchenko, P.V., Plachetka, A., Alekseyenko, A.A., Kuroda, M.I., and Park, P.J. (2013). Comment on "Drosophila dosage compensation involves enhanced Pol II recruitment to male X-linked promoters. Science *340*, 273. https://doi.org/10.1126/science.1231815.
26. Straub, T., and Becker, P.B. (2013). Comment on "Drosophila dosage compensation involves enhanced Pol II recruitment to male X-linked promoters. Science *340*, 273. https://doi.org/10.1126/science.1231895.
27. Vaquerizas, J.M., Cavalli, F.M.G., Conrad, T., Akhtar, A., and Luscombe, N.M. (2013). Response to comments on "Drosophila dosage compensation involves enhanced Pol II recruitment to male X-linked promoters. Science *340*, 273.
28. Rodriguez, J., and Larson, D.R. (2020). Transcription in living cells: molecular mechanisms of bursting. Annu. Rev. Biochem. *89*, 189–212. https://doi.org/10.1146/annurev-biochem-011520-105250.
29. Lott, S.E., Villalta, J.E., Schroth, G.P., Luo, S., Tonkin, L.A., and Eisen, M.B. (2011). Noncanonical compensation of zygotic X transcription in early Drosophila melanogaster development revealed through single-embryo RNA-seq. PLoS Biol. *9*, e1000590. https://doi.org/10.1371/journal.pbio.1000590.
30. Fukaya, T. (2021). Dynamic regulation of anterior-posterior patterning genes in living Drosophila embryos. Curr. Biol. *31*, 2227–2236.e6. https://doi.org/10.1016/j.cub.2021.02.050.

31. Hoppe, C., Bowles, J.R., Minchington, T.G., Sutcliffe, C., Upadhyai, P., Rattray, M., and Ashe, H.L. (2020). Modulation of the promoter activation rate dictates the transcriptional response to graded BMP signaling levels in the Drosophila embryo. Dev. Cell *54*, 727–741.e7. https://doi.org/10.1016/j.devcel.2020.07.007.

32. Stathopoulos, A., and Newcomb, S. (2020). Setting up for gastrulation: D. melanogaster. In Current Topics in Developmental Biology (Elsevier), pp. 3–32. https://doi.org/10.1016/bs.ctdb.2019.11.004.

33. Calvo, L., Ronshaugen, M., and Pettini, T. (2021). smiFISH and embryo segmentation for single-cell multi-gene RNA quantification in arthropods. Commun. Biol. *4*, 352. https://doi.org/10.1038/s42003-021-01803-0.

34. Ferguson, M.L., and Larson, D.R. (2013). Measuring transcription dynamics in living cells using fluctuation analysis. In Imaging Gene Expression Methods in Molecular Biology, Y. Shav-Tal, ed. (Humana Press), pp. 47–60. https://doi.org/10.1007/978-1-62703-526-2_4.

35. Lammers, N.C., Galstyan, V., Reimer, A., Medin, S.A., Wiggins, C.H., and Garcia, H.G. (2020). Multimodal transcriptional control of pattern formation in embryonic development. Proc. Natl. Acad. Sci. USA *117*, 836–847. https://doi.org/10.1073/pnas.1912500117.

36. Larson, D.R., Zenklusen, D., Wu, B., Chao, J.A., and Singer, R.H. (2011). Real-time observation of transcription initiation and elongation on an endogenous yeast gene. Science *332*, 475–478. https://doi.org/10.1126/science.1202142.

37. Fukaya, T., Lim, B., and Levine, M. (2017). Rapid rates of Pol II elongation in the Drosophila embryo. Curr. Biol. *27*, 1387–1391. https://doi.org/10.1016/j.cub.2017.03.069.

38. Garcia, H.G., Tikhonov, M., Lin, A., and Gregor, T. (2013). Quantitative imaging of transcription in living Drosophila embryos links polymerase activity to patterning. Curr. Biol. *23*, 2140–2145. https://doi.org/10.1016/j.cub.2013.08.054.

39. Shermoen, A.W., and O'Farrell, P.H. (1991). Progression of the cell cycle through mitosis leads to abortion of nascent transcripts. Cell *67*, 303–310. https://doi.org/10.1016/0092-8674(91)90182-X.

40. Prudêncio, P., Savisaar, R., Rebelo, K., Martinho, R.G., and Carmo-Fonseca, M. (2022). Transcription and splicing dynamics during early *Drosophila* development. RNA *28*, 139–161. https://doi.org/10.1261/rna.078933.121.

41. Bowles, J.R., Hoppe, C., Ashe, H.L., and Rattray, M. (2022). Scalable inference of transcriptional kinetic parameters from MS2 time series data. Bioinformatics *38*, 1030–1036. https://doi.org/10.1093/bioinformatics/btab765.

42. Zoller, B., Little, S.C., and Gregor, T. (2018). Diverse spatial expression patterns emerge from unified kinetics of transcriptional bursting. Cell *175*, 835–847.e25. https://doi.org/10.1016/j.cell.2018.09.056.

43. Jaeger, J. (2011). The gap gene network. Cell. Mol. Life Sci. *68*, 243–274. https://doi.org/10.1007/s00018-010-0536-y.

44. Ashe, H.L., Mannervik, M., and Levine, M. (2000). Dpp signaling thresholds in the dorsal ectoderm of the Drosophila embryo. Development *127*, 3305–3312.

45. Brouwer, I., and Lenstra, T.L. (2019). Visualizing transcription: key to understanding gene expression dynamics. Curr. Opin. Chem. Biol. *51*, 122–129.

46. Garcia, G.R., and Larson, D.R. (2020). Transcriptional decoding of morphogen gradients during development. Dev. Cell *54*, 687–688.

47. Bothma, J.P., Garcia, H.G., Esposito, E., Schlissel, G., Gregor, T., and Levine, M. (2014). Dynamic regulation of eve stripe 2 expression reveals transcriptional bursts in living Drosophila embryos. Proc. Natl. Acad. Sci. USA *111*, 10598–10603. https://doi.org/10.1073/pnas.1410022111.

48. Berrocal, A., Lammers, N.C., Garcia, H.G., and Eisen, M.B. (2020). Kinetic sculpting of the seven stripes of the Drosophila even-skipped gene. Elife *9*, e61635. https://doi.org/10.7554/eLife.61635.

49. Sun, X.-M., Bowman, A., Priestman, M., Bertaux, F., Martinez-Segura, A., Tang, W., Whilding, C., Dormann, D., Shahrezaei, V., and Marguerat, S. (2020). Size-dependent increase in RNA polymerase II initiation rates mediates gene expression scaling with cell size. Curr. Biol. *30*, 1217–1230.e7. https://doi.org/10.1016/j.cub.2020.01.053.

50. Bernstein, M., and Cline, T.W. (1994). Differential effects of Sex-lethal mutations on dosage compensation early in Drosophila development. Genetics *136*, 1051–1061. https://doi.org/10.1093/genetics/136.3.1051.

51. Gergen, J.P. (1987). Dosage compensation in Drosophila: evidence that *daughterless* and *sex-lethal* control *X* chromosome activity at the blastoderm stage of embryogenesis. Genetics *117*, 477–485. https://doi.org/10.1093/genetics/117.3.477.

52. Kelley, R.L., Solovyeva, I., Lyman, L.M., Richman, R., Solovyev, V., and Kuroda, M.I. (1995). Expression of Msl-2 causes assembly of dosage compensation regulators on the X chromosomes and female lethality in Drosophila. Cell *81*, 867–877. https://doi.org/10.1016/0092-8674(95)90007-1.

53. Cline, T.W. (2005). Reflections on a path to sexual commitment. Genetics *169*, 1179–1185. https://doi.org/10.1093/genetics/169.3.1179.

54. Shi, X., Yang, H., and Birchler, J.A. (2023). MicroRNAs play regulatory roles in genomic balance. Bioessays *45*, 2200187. https://doi.org/10.1002/bies.202200187.

55. Deng, X., Berletch, J.B., Ma, W., Nguyen, D.K., Hiatt, J.B., Noble, W.S., Shendure, J., and Disteche, C.M. (2013). Mammalian X upregulation is associated with enhanced transcription initiation, RNA half-life, and MOF-mediated H4K16 acetylation. Dev. Cell *25*, 55–68. https://doi.org/10.1016/j.devcel.2013.01.028.

56. Forbes Beadle, L., Love, J.C., Shapovalova, Y., Artemev, A., Rattray, M., and Ashe, H.L. (2023). Combined modelling of mRNA decay dynamics and single-molecule imaging in the Drosophila embryo uncovers a role for P-bodies in 5′ to 3′ degradation. PLoS Biol. *21*, e3001956. https://doi.org/10.1371/journal.pbio.3001956.

57. Pimmett, V.L., Dejean, M., Fernandez, C., Trullo, A., Bertrand, E., Radulescu, O., and Lagha, M. (2021). Quantitative imaging of transcription in living Drosophila embryos reveals the impact of core promoter motifs on promoter state dynamics. Nat. Commun. *12*, 4504. https://doi.org/10.1038/s41467-021-24461-6.

58. Tantale, K., Garcia-Oliver, E., Robert, M.-C., L'Hostis, A., Yang, Y., Tsanov, N., Topno, R., Gostan, T., Kozulic-Pirher, A., Basu-Shrivastava, M., et al. (2021). Stochastic pausing at latent HIV-1 promoters generates transcriptional bursting. Nat. Commun. *12*, 4503. https://doi.org/10.1038/s41467-021-24462-5.

59. Batut, P.J., Bing, X.Y., Sisco, Z., Raimundo, J., Levo, M., and Levine, M.S. (2022). Genome organization controls transcriptional dynamics during development. Science *375*, 566–570. https://doi.org/10.1126/science.abi7178.

60. Levo, M., Raimundo, J., Bing, X.Y., Sisco, Z., Batut, P.J., Ryabichko, S., Gregor, T., and Levine, M.S. (2022). Transcriptional coupling of distant regulatory genes in living embryos. Nature *605*, 754–760. https://doi.org/10.1038/s41586-022-04680-7.

61. Kaye, E.G., Booker, M., Kurland, J.V., Conicella, A.E., Fawzi, N.L., Bulyk, M.L., Tolstorukov, M.Y., and Larschan, E. (2018). Differential occupancy of two GA-binding proteins promotes targeting of the Drosophila dosage compensation complex to the male X chromosome. Cell Rep. *22*, 3227–3239. https://doi.org/10.1016/j.celrep.2018.02.098.

62. Collombet, S., Rall, I., Dugast-Darzacq, C., Heckert, A., Halavatyi, A., Le Saux, A., Dailey, G., Darzacq, X., and Heard, E. (2021). RNA polymerase II depletion from the inactive X chromosome territory is not mediated by physical compartmentalization. Preprint at bioRxiv. https://doi.org/10.1101/2021.03.26.437188.

63. Senecal, A., Munsky, B., Proux, F., Ly, N., Braye, F.E., Zimmer, C., Mueller, F., and Darzacq, X. (2014). Transcription factors modulate c-fos transcriptional bursts. Cell Rep. *8*, 75–83. https://doi.org/10.1016/j.celrep.2014.05.053.

64. Falo-Sanjuan, J., Lammers, N.C., Garcia, H.G., and Bray, S.J. (2019). Enhancer priming enables fast and sustained transcriptional responses to Notch signaling. Dev. Cell *50*, 411–425.e8. https://doi.org/10.1016/j.devcel.2019.07.002.

65. Lee, C., Shin, H., and Kimble, J. (2019). Dynamics of notch-dependent transcriptional bursting in its native context. Dev. Cell *50*, 426–435.e4. https://doi.org/10.1016/j.devcel.2019.07.001.

66. Larsson, A.J.M., Johnsson, P., Hagemann-Jensen, M., Hartmanis, L., Faridani, O.R., Reinius, B., Segerstolpe, Å., Rivera, C.M., Ren, B., and Sandberg, R. (2019). Genomic encoding of transcriptional burst kinetics. Nature *565*, 251–254. https://doi.org/10.1038/s41586-018-0836-1.

67. Hunt, G.J., Vaid, R., Pirogov, S., Pfab, A., Ziegenhain, C., Sandberg, R., Reimegård, J., and Mannervik, M. (2022). Comprehensive interrogation of a Drosophila embryonic patterning network reveals the impact of chromatin state on tissue-specific burst kinetics and RNA Polymerase II promoter-proximal pause release. Preprint at bioRxiv. https://doi.org/10.1101/2022.10.25.513691.

68. Calderon, D., Blecher-Gonen, R., Huang, X., Secchia, S., Kentro, J., Daza, R.M., Martin, B., Dulja, A., Schaub, C., Trapnell, C., et al. (2022). The continuum of *Drosophila* embryonic development at single-cell resolution. Science *377*, eabn5800. https://doi.org/10.1126/science.abn5800.

69. Ing-Simmons, E., Vaid, R., Bing, X.Y., Levine, M., Mannervik, M., and Vaquerizas, J.M. (2021). Independence of chromatin conformation and gene regulation during Drosophila dorsoventral patterning. Nat. Genet. *53*, 487–499. https://doi.org/10.1038/s41588-021-00799-x.

70. Tsanov, N., Samacoits, A., Chouaib, R., Traboulsi, A.-M., Gostan, T., Weber, C., Zimmer, C., Zibara, K., Walter, T., Peter, M., et al. (2016). smiFISH and FISH-quant – a flexible single RNA detection approach with super-resolution capability. Nucleic Acids Res. *44*, e165. https://doi.org/10.1093/nar/gkw784.

71. Bertrand, E., Chartrand, P., Schaefer, M., Shenoy, S.M., Singer, R.H., and Long, R.M. (1998). Localization of ASH1 mRNA particles in living yeast. Mol. Cell *2*, 437–445. https://doi.org/10.1016/s1097-2765(00)80143-4.

72. Gratz, S.J., Cummings, A.M., Nguyen, J.N., Hamm, D.C., Donohue, L.K., Harrison, M.M., Wildonger, J., and O'Connor-Giles, K.M. (2013). Genome engineering of Drosophila with the CRISPR RNA-guided Cas9 nuclease. Genetics *194*, 1029–1035. https://doi.org/10.1534/genetics.113.152710.

73. Gratz, S.J., Ukken, F.P., Rubinstein, C.D., Thiede, G., Donohue, L.K., Cummings, A.M., and O'Connor-Giles, K.M. (2014). Highly specific and efficient CRISPR/Cas9-catalyzed homology-directed repair in Drosophila. Genetics *196*, 961–971. https://doi.org/10.1534/genetics.113.160713.

74. Hoppe, C., and Ashe, H.L. (2021). CRISPR-Cas9 strategies to insert MS2 stem-loops into endogenous loci in Drosophila embryos. STAR Protoc. *2*, 100380. https://doi.org/10.1016/j.xpro.2021.100380.

75. Kosman, D., Mizutani, C.M., Lemons, D., Cox, W.G., McGinnis, W., and Bier, E. (2004). Multiplex detection of RNA expression in *Drosophila* embryos. Science *305*, 846. https://doi.org/10.1126/science.1099247.

76. Gloor, G.B., Preston, C.R., Johnson-Schlitz, D.M., Nassif, N.A., Phillis, R.W., Benz, W.K., Robertson, H.M., and Engels, W.R. (1993). Type I repressors of P element mobility. Genetics *135*, 81–95. https://doi.org/10.1093/genetics/135.1.81.

77. Hoppe, C., and Ashe, H.L. (2021). Live imaging and quantitation of nascent transcription using the MS2/MCP system in the Drosophila embryo. STAR Protoc. *2*, 100379. https://doi.org/10.1016/j.xpro.2021.100379.

STAR★METHODS

KEY RESOURCES TABLE

REAGENT or RESOURCE	SOURCE	IDENTIFIER
Antibodies		
Goat anti-Mouse IgG (H + L) Cross-Adsorbed Secondary Antibody, Alexa Fluor 488	Thermo Fisher Scientific	Cat# A-11001; RRID: AB_2534069
Spectrin, alpha antibody	Developmental Studies Hybridoma Bank	Cat# 3A9 (323 or M10-2); RRID: AB_528473
Chemicals, peptides, and recombinant proteins		
Halocarbon oil 27	Sigma	Cat# H8773; CAS: 9002-83-9
Halocarbon oil 700	Sigma	Cat# H8898; CAS: 9002-83-9
DAPI	New England Biolabs	Cat# 4083; CAS: 28718-90-3
ProLong^{TM} Diamond Antifade Mountant	Thermo Fisher Scientific	Cat# P36961
Western Blocking Reagent, Solution	Sigma	Cat# 11921673001
Experimental models: Organisms/strains		
D. melanogaster; *y^{1}w^{67c23}*	Bloomington Drosophila Stock Center	RRID:BDSC_6599
D. melanogaster; His2Av-mRFP; nos-MCP-eGFP	Bloomington Drosophila Stock Center, Garcia et al.[38]	RRID:BDSC_60340
D. melanogaster; *w1118 sog-MS2*	This Study	N/A
D. melanogaster; *w1118 mew-MS2*	This Study	N/A
D. melanogaster; *y^{1}M{vas-Cas9}ZH-2Aw^{1118}*, *24xMS2-hnt*;	Hoppe et al.[31]	N/A
D. melanogaster; *gt-MS2*	Fukaya.[30]	N/A
D. melanogaster; y1w67c23; MKRS, P{hsFLP}86E/TM6B, P{Crew}DH2, Tb1	Bloomington Drosophila Stock Center	RRID:BDSC_1501
D. melanogaster; *w^{1118}*; *PBac{vas-Cas9}VK00027*	Bloomington Drosophila Stock Center	RRID:BDSC_51324
Oligonucleotides		
smiFISH probes	Merck	This study
Quasar® 670 Z-FLAP CCAGCTTCTAGCATCCATGCCCTATAAG	Biosearch Technologies; 2BScientific (Tsanov et al.[70])	N/A
Quasar® 570 X-FLAP CCTCCTAAGTTTC GAGCTGGACTCAGTG	Biosearch Technologies; 2BScientific (Tsanov et al.[70])	N/A
Recombinant DNA		
pCR4-24XMS2SL-stable	Addgene (Bertrand et al.[71])	RRID: Addgene_31865
pU6-BbsI-chiRNA	Addgene (Gratz et al.[72])	RRID:Addgene_45946
pHD-DsRed	Addgene (Gratz et al.[73])	RRID:Addgene_51434
Software and algorithms		
FIJI (ImageJ)	NIH	RRID:SCR_002285
Imaris $\geq$9.2	Bitplane	RRID:SCR_007370
GraphPad Prism 9.1.2	GraphPad Software	RRID: SCR_002798
R 4.1.2	The R Foundation	https://www.r-project.org/
Huygens Professional Deconvolution	SVI (Scientific Volume Imaging)	RRID:SCR_014237 https://svi.nl/Huygens-Professional
Simple Assignment of Spots to Surfaces Python Script	Hoppe et al.[31]	https://github.com/TMinchington/sass
Memory-adjusted hidden Markov model for single cell parameters	Bowles et al.[41]	https://github.com/ManchesterBioinference/burstInfer

(*Continued on next page*)

Continued		
REAGENT or RESOURCE	SOURCE	IDENTIFIER
Other		
Leica TCS SP8 AOBS inverted microscope	Leica	N/A
Andor Dragonfly200 spinning disk upright confocal microscope with inbuilt deconvolution	Andor (Oxford Instruments)	N/A
lumox® dish 50, Cell Culture Dish	Sarstedt AG & Co	Cat# 94.6077.305
Coverslips No 1 18 × 18mm	Scientific Laboratory Supplies	Cat# MIC3110
Coverslips No 0 18 × 18mm	Scientific Laboratory Supplies	Cat# MIC3100
Coverslips No 1 24 × 40mm	Scientific Laboratory Supplies	Cat# MIC3252
Wheaton vials	Sigma	Cat# Z115053-12EA
Phusion® High-Fidelity DNA Polymerase	New England Biolabs	Cat# M0530
GoTaq® Master Mix	Promega	Cat# M7123
PfuTurbo DNA Polymerase	Agilent	Cat# 600250
1kb Plus DNA Ladder	Thermo Fisher Scientific	Cat# 10787018

RESOURCE AVAILABILITY

Lead contact

Further information and requests for resources and reagents should be directed to and will be fulfilled by the lead contact, Hilary L. Ashe (hilary.ashe@manchester.ac.uk).

Materials availability

Plasmids and fly lines generated in this study are available without restriction from the lead contact on request.

Data and code availability

- Data reported in this paper will be shared by the lead contact upon request.
- This paper does not report original code.
- Any additional information required to reanalyze the data reported in this paper is available from the lead contact upon request.

EXPERIMENTAL MODEL AND SUBJECT DETAILS

Experimental animals and crosses

All stocks were grown and maintained at 20°C and raised at 25°C for experiments on standard fly food media (yeast 50 g/L, glucose 78 g/L, maize 72 g/L, agar 8 g/L, 10% nipagen in EtOH 27 mL/L and propionic acid 3 mL/L).

The following fly lines were used in this study, y^1 w^{67c23} (BDSC Stock #6599, RRID:BDSC_6599), y^1 w^*; P{His2Av-mRFP1}II.2; P{nos-MCP.EGFP}2 (BDSC Stock #60340, RRID:BDSC_60340), y^1 w^{1118} M{vas-Cas9}ZH-2A; 24xMS2-*hnt*,[31] *gtMS2*,[30] w^{1118} 24xMS2-*sog* (this study), w^{1118} 24xMS2-*mew* (this study), w^{1118}; PBac{vas-Cas9}VK00027 (BDSC Stock #51324, RRID:BDSC_51324), y^1 w^{67c23}; MKRS, P{ry[+t7.2] = hsFLP86E/TM6B, P{w[+mC] = Crew}DH2, Tb^1 (BDSC Stock #1501, RRID:BDSC_1501).

For all live imaging experiments His2Av-mRFP; nos-MCP-EGFP virgin females were crossed to males carrying the target gene-MS2 locus. F1 virgin females of genotype gene-MS2/+; His2Av-mRFP/+; nos-MCP-EGFP/+ were crossed to gene-MS2 males to obtain F2 male and female embryos (Figure 1B) containing the gene-MS2 locus and maternally loaded His2Av-RFP and MCP-EGFP. The male and female F2 embryos analyzed have one copy of the gene-MS2 insertion.

METHOD DETAILS

CRISPR of 24X MS2 loops into endogenous loci

24xMS2 loops[71] (from pCR4-24XMS2SL-stable, RRID: Addgene_31865) were inserted into the first intron of *sog* and *mew* using two guide RNAs and one-step CRISPR Cas9 genome engineering.[72–74] Briefly, two guide regions were chosen within the first intron of the *sog* and *mew* genomic loci at a central position to avoid splice sites. A double stranded donor plasmid was constructed containing the intronic region that was removed between the two guides, the 24xMS2 loop cassette and a dsRed marker (from pHD-DsRed, RRID:Addgene_51434) inserted using a ClaI site for *mew* and AccII site for *sog*. The PAM sequences within each donor plasmid

were mutated using site directed mutagenesis with Pfu Turbo (Agilent, Cat# 600250) to avoid targeting of the donor plasmid by Cas9 nuclease. Both the donor plasmid and the two guide RNA plasmids (pU6-BbsI-chiRNA, RRID:Addgene_45946) for each gene were injected into Cas9 embryos (BDSC Stock #51324, RRID:BDSC_51324) by the Cambridge Fly Facility. Oligonucleotide sequences for guide RNAs are listed in Table S1. Successful transformants were selected using the dsRed marker, which was subsequently removed by crossing to a Cre recombinase stock (BDSC Stock #1501, RRID:BDSC_1501) and screening for loss of the marker in the next generation. All primer sequences are listed in Table S1.

Single molecule fluorescent *in situ* hybridisation

2-4 h embryos were fixed as previously described[75] and stored in methanol at -20°C until required. Fixed embryos were placed in Wheaton vials (Sigma, Cat# Z115053-12EA) for FISH as described previously.[31] Embryos were probed for the mRNA target using smiFISH fluorescent probes designed to exonic sequences of *gt*, *sog* and *mew* with X or Z flap sequences[70] and secondary detection probes labeled with Quasar 570 or 670 fluorophore (all probe sequences are listed in Table S1). Mouse α-Spectrin antibody (1:50 DSHB, 3A9 (323 or M10-2), RRID:AB_528473) incubation overnight at 4°C was used with a secondary Goat anti-Mouse IgG (H + L) Cross-Adsorbed Secondary Antibody, Alexa Fluor 488 (1:1000 Thermo Fisher Scientific, Cat# A-11001, RRID:AB_2534069) for 2 h at room temperature to stain the membrane. DAPI (New England Biolabs, Cat# 4083) was added to the embryos in the second of the final four washes of the protocol at a concentration of 1:1000 and embryos were mounted onto slides in Prolong Diamond (Thermo Fisher Scientific, Cat# P36961) to set overnight before imaging.

PCR assay to identify embryo sex

After live-imaging, individual embryos were carefully picked off the imaging dish and stored at -20°C in individual tubes. DNA was extracted from single embryos by crushing them in 50μL of squishing buffer (10mM Tris-Cl pH8.2, 1mM EDTA, 25mM NaCl, 200ug/mL Proteinase K) and incubating at 25°C for 25 min followed by 2 min incubation at 95°C to inactivate the Proteinase K.[76] PCR amplification of DNA was performed using GoTaq (Promega, Cat# M7123) or Phusion (New England Biolabs, Cat# M0530) DNA polymerase following the manufacturer's protocol. Primers were used that flanked the 24xMS2 cassette insertion and amplified the *kl-5* gene on the Y chromosome (primer sequences are listed in Table S1), which allowed detection of the presence of the MS2 loops on the X chromosome and either an unmodified locus (female embryos) or the *kl-5* gene (male embryos). Due to the repetitive nature of the MS2 loops, the primers that flank the MS2 insertion produce a PCR product that can vary from ~1 to 1.6kb therefore the PCR product from the unmodified locus in combination with the male Y chromosome band was used primarily to identify embryo sex. PCR reactions were performed in triplicate for each embryo. The 1 kb Plus DNA Ladder (Thermo Fisher Scientific, Cat# 10787018) was used in Figure 1 with the following band sizes shown on the gel (100, 200, 300, 400, 500, 650, 850, 1000, 1500bp).

Viability assay

y w/y^{+} *w sogMS2* females were crossed to *y*$^{+}$ *w sogMS2/Y* males. Each replicate experiment consisted of six vials, each containing thirty larvae. The survival of adult males was measured by calculating the relative proportion of *y* and *y*$^{+}$ males emerging. The same crossing scheme was used to assess *mewMS2* viability.

Confocal microscopy of fixed embryos

An Andor Dragonfly200 spinning disk upright confocal microscope with a 40x/1.30 HCL pL Apochromat objective was used to acquire smFISH images of *sog* and *mew* in fixed embryos. Samples were excited using 405nm (10%), 488nm (11%) and 637nm (10%) diode lasers respectively. Images were collected with an iXon EMCCD camera (1024 X 1024) with a gain of 180 for 130ms of multiple Z stacks at system optimised spacing.

For experiments quantifying *gt* mRNA counts (Figure S1E) a Leica TCS SP8 gSTED confocal was used with a 100x/1.3 HC Pl Apo Cs2 objective at 0.75× zoom. Confocal settings were 1 airy unit pinhole, 400 Hz scan speed with bidirectional line scanning and a format of 4096 × 4096 pixels. Laser detection settings were as follows: PMT detector DAPI excitation at 405nm (7%, collection: 415-470nm); Hybrid SMD Detectors: Alexa Fluor 488 excitation at 490nm (12%, collection: 500-540nm), Quasar 570 excitation at 548nm (20%, collection: 558-640nm) with 1-6ns gating. All images were collected sequentially and optical stacks were acquired at system optimised spacing. Imaging of the membrane stained with anti-Spectrin at the mid-sagittal plane of the embryo with 40× objective at 0.75× zoom and 1024 X 1024 format was used to measure the average length of membrane invagination from at least 5 cells. These measurements were used to select embryos of a similar age in early nuclear cycle 14 (~5μm membrane invagination). 6 separate embryos of each sex were imaged and quantified as independent replicates.

Live imaging microscopy

Embryos were laid on apple juice agar plates for approximately 1 h and embryos were collected and dechorionated in 50% bleach solution (2.5% final concentration of sodium hypochlorite solution diluted in water). Preparation of embryos for live imaging was performed as described,[77] with embryos mounted onto a heptane glue coated coverslip (Scientific Laboratory Supplies, Cat# MIC3110) and inverted over a coverslip bridge in a 7:1 ratio mix of 700:27 halocarbon oil (Sigma, Cat# H8773 and Cat# H8898) on the membrane of a Lumox dish (Sarstedt AG & Co, Cat# 94.6077.305). Images were collected on an Andor Dragonfly200 spinning disk upright confocal microscope with a 40x/1.30 HCL pL Apochromat objective. Samples were excited using 488nm (11%; *sogMS2*,

gtMS2 and *mewMS2* or 13%; *hntMS2*) and 561nm (6%) diode lasers via Leica GFP and RFP filters respectively. Images were collected simultaneously using dual camera imaging with Zyla 4.2 Plus sCMOS (2048 X 2048) and iXon EMCCD camera (1024 X 1024) with a gain of 180 and binning [2X and 1X respectively] for 130ms. For each movie a total of 50 Z stacks at 0.7μm spacing were collected using the fastest setting yielding a total Z size of 35 μm at a time resolution of between 20 and 25 s on average.

Image deconvolution

Images were deconvolved using either the inbuilt Andor deconvolution software for the live embryo movies or Huygens professional deconvolution software by SVI (Scientific Volume Imaging, RRID:SCR_014237) for smFISH images. smFISH images of whole embryos were tiled using the Grid/Collection stitching plugin in FIJI (ImageJ) (NIH, RRID: SCR 002285).

QUANTIFICATION AND STATISTICAL ANALYSIS

Live and fixed embryo image analysis

Imaris software ≥9.2.1 (Bitplane, RRID:SCR_007370) was used for nuclear segmentation and spot detection of transcription sites (TSs) in live imaging movies. Nuclear segmentation was performed using the "surface" function with tracking autoregressive motion and maximum frame gap of 5 and travel distance of 5μm. The "spots" function was used to detect TSs in 3D with a set size of 1.5μm in X/Y diameter and 5μm (*gtMS2)* or 10μm (*sogMS2*, *mewMS2* and *hntMS2)* in the Z direction. Multiple background spots of the same size as the TS were added manually to every third time point to be used for background correction of the fluorescent signal. For fixed embryos the same nuclear segmentation (without tracking) and spot detection was used with spots of size 0.2 μm used to detect single mRNAs instead of TSs. All statistics were exported and the custom sass python script assigned the TS spots to nuclei across time with background correction or mRNA spots to nuclei at a single time point (Github; https://github.com/TMinchington/sass). Further statistical and data analysis was performed in R (version 4.1.2), Python and GraphPad Prism (9.1.2, RRID: SCR 002798). For all instances where nuclei were binned, bins used were 5μm in width (approximately one nucleus in width).

Autocorrelation estimation of elongation time

To determine the rate of Pol II elongation we used the data from the MS2 movies and determined the autocorrelation function of fluorescent traces[36] known as G(τ)

$$G(\tau) = \frac{(T - \tau)}{cT^2} H(T - \tau),$$

where T is the dwell time, τ is the autocorrelation delay, c is the initiation rate of Pol II and H is the Heaviside step function. This function calculates the degree to which a fluorescence signal at one time point $F(t)$ is correlated with a lagged signal of itself $F(t - \tau)$ as a function of τ. Therefore the fluorescence signal at any given time point t will be correlated with an earlier fluorescence value $F(t - \tau)$ when $\tau < T$. Under this condition, the two time points will have shared Pol II on the gene and will therefore be correlated. As τ increases, the correlation from the shared Pol II on the gene will decline linearly until it reaches a transition point at which point it will be equivalent to T and is taken as the time for Pol II to traverse the gene or for splicing to occur. The dwell time was calculated for fluorescence traces individually and the median value for each embryo calculated. For this analysis we used transcriptional traces for each gene with moderate to high transcriptional activity, due to difficulties with the analysis for the sparse traces from low expressing nuclei. The elongation rate for *hnt* was calculated by dividing the gene length by the dwell time.

Modeling transcriptional parameters

MS2 fluorescence traces from all nuclei were used to infer promoter states using a memory-adjusted hidden Markov model (mHMM) implemented in python with a truncated state-space approximation.[41] The model was trained on each embryo separately to generate the transcriptional parameters by sex. The global parameters obtained were the rate of promoter switching on (k_{on}) and off (k_{off}), the Pol II initiation rate (k_{ini}) and promoter mean occupancy as defined previously.[42]

Single cell parameters were determined from the mHMM for each embryo.[41] Single cell parameters were combined for all female and male replicates and plotted into 5μm bins across the expression domain for each gene (Figures 4 and 5). For each replicate the single cell parameters were plotted against either the corresponding distance to the expression domain midline/border or mean expression to determine correlations (Figure 6). To determine t_{on} for a given nucleus, the first time a nucleus increases from zero was taken and for t_{off} a nucleus must be reduced to zero for 5 consecutive time points at the end of the trace. If a nucleus did not have a t_{off} due to transcription still being active at the end of the imaging period, then the final imaging time point was taken as t_{off}. All statistical analysis was carried out in R (version 4.1.2), Python and GraphPad Prism (9.1.2, RRID: SCR 002798).

Cell Reports, Volume *42*

Supplemental information

Modulation of transcription burst amplitude underpins dosage compensation in the *Drosophila* embryo

Lauren Forbes Beadle, Hongpeng Zhou, Magnus Rattray, and Hilary L. Ashe

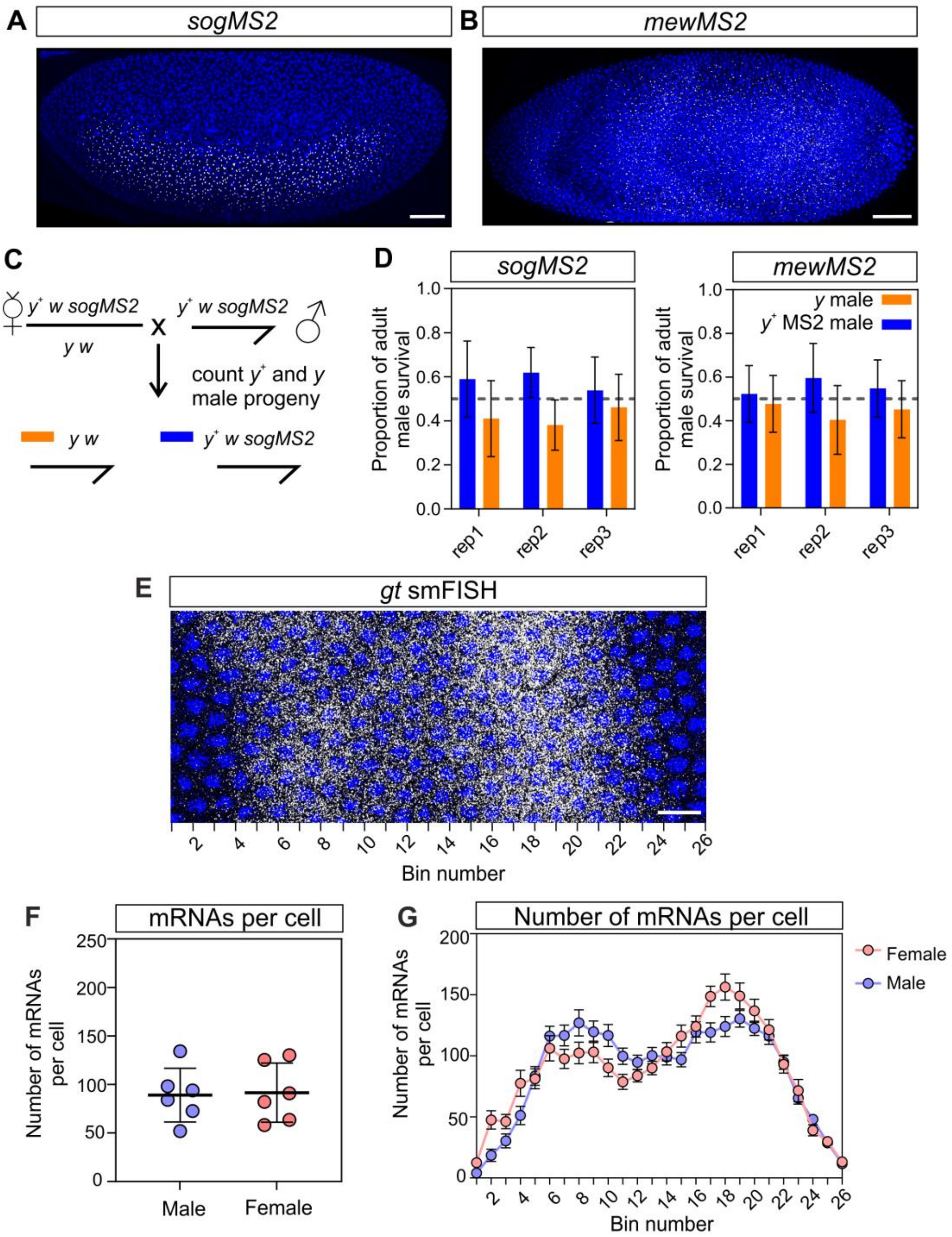

Figure S1. Viability and smFISH analysis of the MS2 lines. Related to Figures 1 and 2.

(A) smFISH of a *sogMS2* embryo with *sog* probes (grey) and DAPI staining (blue) showing the expected expression domain. A lateral view of the embryo is shown at nc14.

(B) As in (A) except a *mewMS2* embryo is stained with *mew* smFISH probes and a dorsal view is shown.

(C) Overview of the crossing scheme to determine the viability of adult flies containing the *sog* or *mew* locus with a 24xMS2 insertion (*sogMS2* is shown as the example).

(D) Graphs show the viability of *sogMS2* or *mewMS2* males relative to those carrying an unedited X chromosome. The dotted line on the graph marks the expected proportion if viability is unaffected by insertion of the MS2 loops. Differences are not significant between control and MS2 male survival.

$p > 0.05$, n= 5-6 independent experiments for each of 3 biological replicates, Holm-Sidak's multiple t-test. Mean ± SD of n = 87, 86 and 98 (*sog*MS2 males) and n = 68, 84 and 63 (*mew*MS2 males).

(E) Representative smFISH image showing *gt* mRNAs in part of a nc14 embryo, with the expression domain divided into different bins.

(F) Graph shows the mean number of *gt* mRNAs/cell for male or female embryos. Mean ± SD of n = 6 embryos of each sex.

(G) Graph shows the mean number of mRNAs per cell binned spatially across the expression domain at early nc14, bin numbers are as in (E). Mean ± SEM of all cells within the binned region, typically 50 cells/bin. n= 6 embryos of each sex.

Scale bar is 50µm in A and B and 10µm in E.

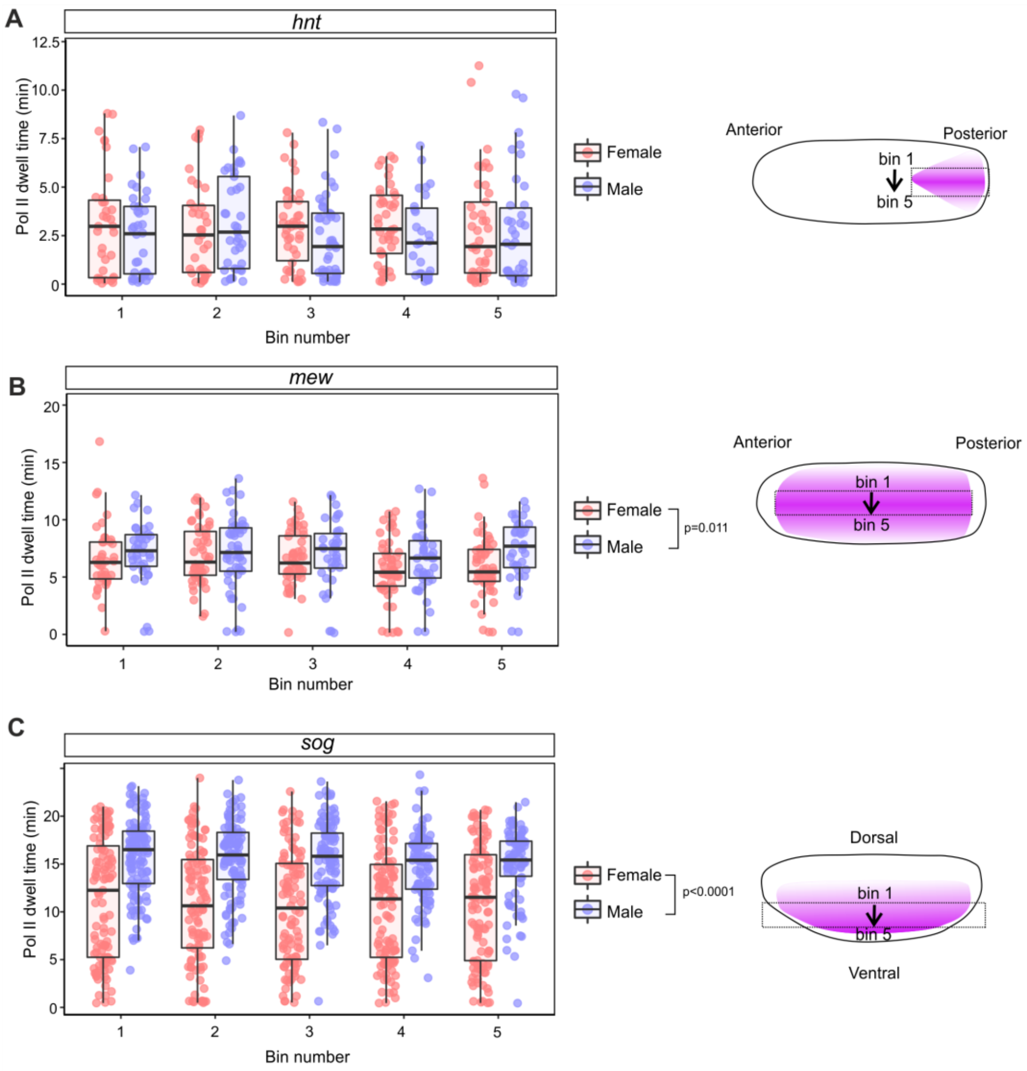

Figure S2. Spatial analysis of Pol II dwell times in embryos. Related to Figure 3.

(A-C) Boxplots of the Pol II/MS2 fluorescence dwell time per transcription site plotted for each bin across a region of the expression domain for (A) *hnt*, (B) *mew* and (C) *sog* in male and female embryos. Boxes show 25th to 75th percentile, line shows median and whiskers show 1.5x interquartile range. Bin numbers correspond to those in the cartoons. A two way ANOVA was used to determine statistical significance. Testing the effect of sex and bin number on Pol II dwell time found that males had a significantly longer Pol II dwell time for *sog* $F_{1,\ 1039} = 194.6$, $p < 0.0001$ and *mew* $F_{1,\ 439} = 6.5$, $p = 0.0114$ but not for *hnt.* There was no significant effect of the bin number on Pol II dwell time for any of the genes, consistent with no spatial regulation of elongation rate. n=194 (*hnt* female) and n=187 (*hnt* male), n=247 (*mew* female) and n=202 (*mew* male), n= 533 (*sog* female) and n=516 (*sog* male) from 3 biological replicates of each sex.

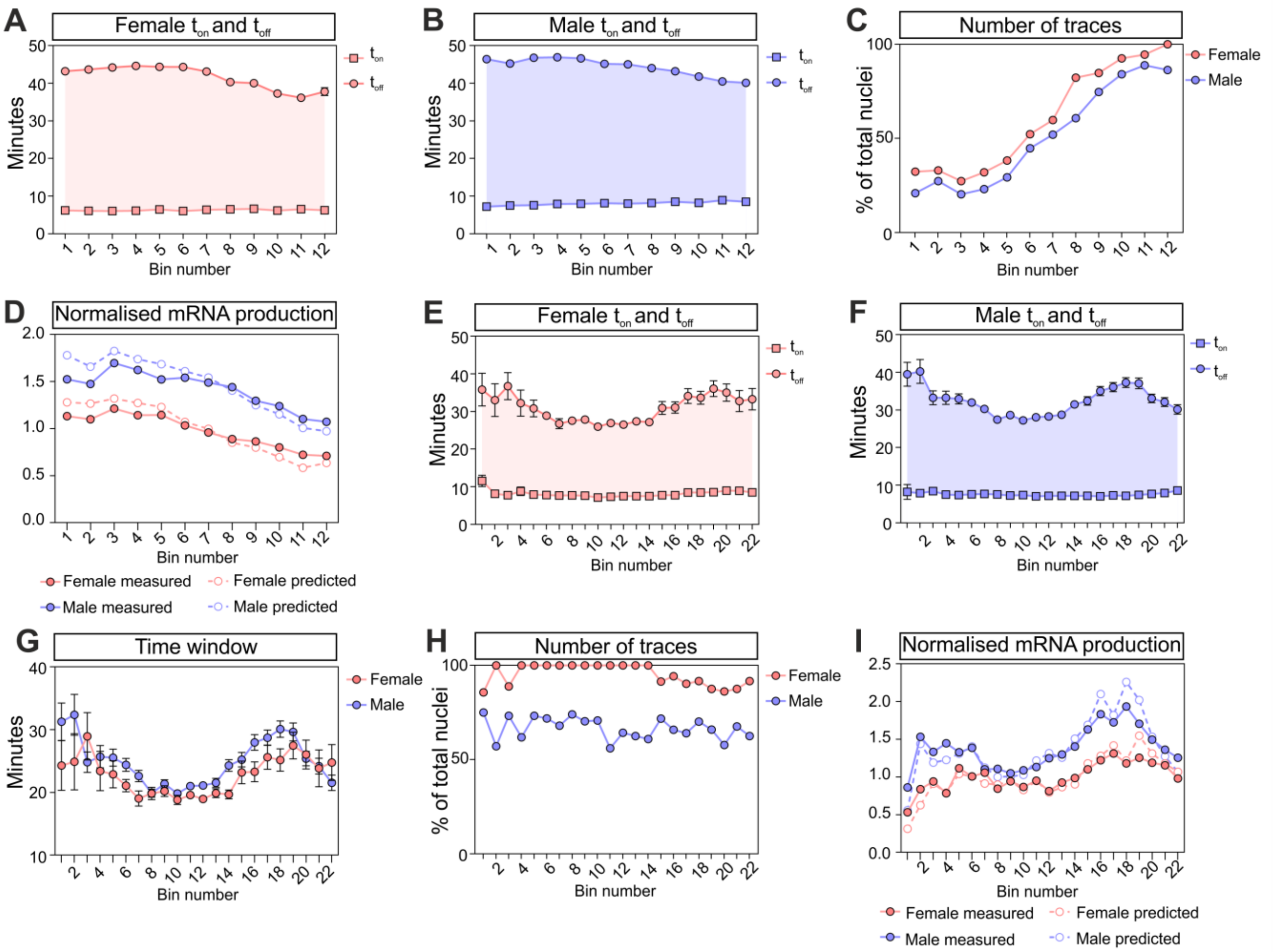

Figure S3. The *sog* and *gt* transcription time windows in male and female embryos. Related to Figures 4 and 5.

(A, B) Graphs show the mean values for t_{on} (squares) and t_{off} (circles) in nuclei from *sogMS2* female (A) and male (B) embryos. The single cell bins are as described in Figure 4B. The shaded area represents the total time window (t_{off} - t_{on}).

(C) Percentage of *sogMS2* nuclear traces that have a t_{off} within the imaging period.

(D) Graph shows the predicted total *sogMS2* expression in male and female embryos, based on multiplying Pol II initiation rate, the active time window and promoter occupancy (open circles, dashed lines) with the expression data from Figure 4C plotted for comparison (closed circles). Data have been normalised to the mean value from the female data.

(E, F). As in (A, B), except the data are for *gtMS2*. The single cell bins are as described in Figure 5A.

(G) Graph shows the mean time window estimated for *gtMS2* across single cell bins in male and female embryos.

(H) As in (C), except the data are for *gtMS2*.

(I) As in (D), except the data are for *gtMS2.*

Mean ± SEM, n= 1076 (*sog* female) and 1081 (*sog* male), n= 437 (*gt* female) and 675 nuclei (*gt* male) pooled from 3 biological replicates for each sex.

A

Replicate	k_{on}	k_{off}	Occupancy	Initiation rate
sog female 1	0.55	-0.75	0.91	0.17
sog female 2	0.84	-0.67	0.99	0.05
sog female 3	0.70	-0.71	0.98	0.18
sog male 1^	0.74	-0.67	0.99	0.07
sog male 2	0.81	-0.77	0.97	0.07
sog male 3	0.68	-0.63	0.99	-0.08

B

Replicate	k_{on}	k_{off}	Occupancy	Initiation rate
gt female 1	0.64	-0.34	0.88	0.38
gt female 2	0.60	-0.43	0.93	0.20
gt female 3	0.63	-0.42	0.90	0.59
gt male 1^	0.70	-0.48	0.89	0.66
gt male 2	0.74	-0.32	0.87	0.57
gt male 3	0.66	-0.34	0.89	0.66

C *gt*

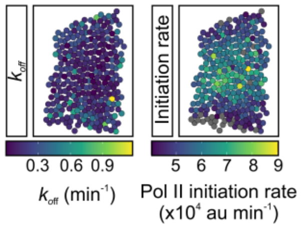

D

Replicate	k_{on}	k_{off}	Occupancy	Initiation rate
hnt female 1	0.89	-0.32	0.99	0.15
hnt female 2	0.88	-0.46	0.99	0.05
hnt female 3	0.85	-0.29	0.99	0.14
hnt male 1^	0.89	-0.21	0.99	-0.11
hnt male 2	0.87	-0.28	0.99	-0.12
hnt male 3	0.82	-0.53	0.99	0.04

E *hnt*

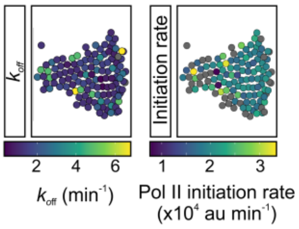

F *mew*

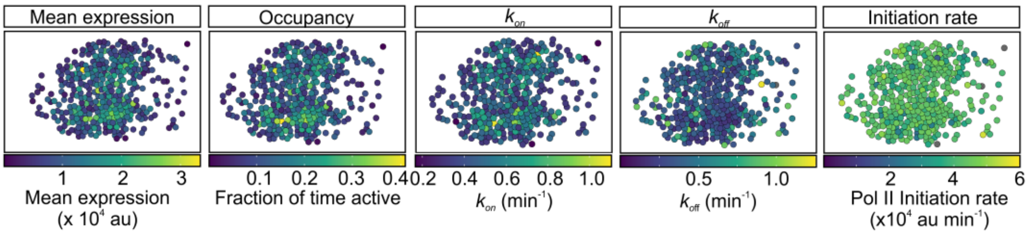

G

Replicate	k_{on}	k_{off}	Occupancy	Initiation rate
mew female 1	0.65	-0.39	0.97	0.18
mew female 2	0.50	-0.56	0.97	0.37
mew female 3	0.64	-0.51	0.95	0.34
mew male 1^	0.62	-0.48	0.96	0.12
mew male 2	0.66	-0.55	0.97	0.39
mew male 3	0.48	-0.55	0.97	0.31

Figure S4. Correlations between mean expression and single cell burst parameters for all nuclei in the expression domain. Related to Figure 6.

(A, B, D, G) Table shows the Pearson correlation coefficients between mean expression (arbitrary units) and the indicated burst parameter for all nuclei in each individual male and female biological replicate embryo analysed. The correlations are shown for *sog* (A), *gt* (B), *hnt* (D) and *mew* (G). The ^ label denotes the embryo used to show the data as spatial heatmaps in panels S4C, E and F and Figure 6A, C, D.

(C, E) Spatial heatmaps from a representative *gt* (C) and *hnt* (E) embryo showing k_{off} and Pol II initiation rate with nuclei coloured as in the associated key.

(F) Heatmaps from a representative *mew* embryo, with nuclei in the expression domain coloured depending on their value for either mean expression or the indicated burst parameter. The colour keys are shown below each heatmap.
